# Coarse-Grained Modeling Using Neural Networks Trained on Structural Data

**DOI:** 10.1021/acs.jctc.3c00516

**Published:** 2023-09-15

**Authors:** Mikhail Ivanov, Maksim Posysoev, Alexander P. Lyubartsev

**Affiliations:** Department of Materials and Environmental Chemistry, Stockholm University, SE-106 91 Stockholm, Sweden

## Abstract

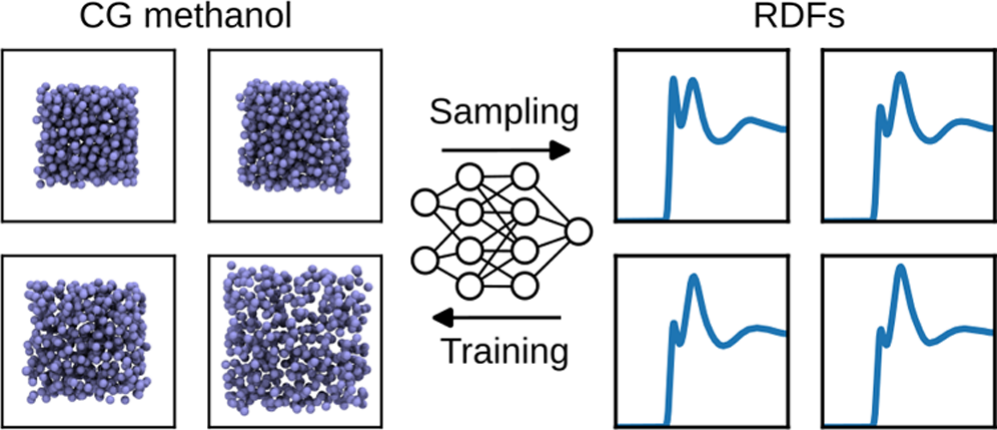

We propose a method of bottom-up coarse-graining, in which interactions within a coarse-grained model are determined by an artificial neural network trained on structural data obtained from multiple atomistic simulations. The method uses ideas of the inverse Monte Carlo approach, relating changes in the neural network weights with changes in average structural properties, such as radial distribution functions. As a proof of concept, we demonstrate the method on a system interacting by a Lennard–Jones potential modeled by a simple linear network and a single-site coarse-grained model of methanol–water solutions. In the latter case, we implement a nonlinear neural network with intermediate layers trained by atomistic simulations carried out at different methanol concentrations. We show that such a network acts as a transferable potential at the coarse-grained resolution for a wide range of methanol concentrations, including those not included in the training set.

## Introduction

Modeling of many phenomena in biomolecular and materials science requires consideration of a wide range of spatial and time scales, not achievable within a single model or simulation method. Development of multiscale methods, connecting models from ab initio computations of electron structure to the mesoscale, was at the center of the molecular modeling methodology in the last decades.^1−4^ Multiscale modeling is closely related to coarse-graining, which is a consecutive reduction of less important degrees of freedom while keeping important ones.^5^ In order to build a coarse-grained (CG) model, one needs to specify molecular groups assigned as CG units and determine the interaction potential (force field) describing interactions between CG sites. Often, such CG models are built by empirical parametrization of the interaction potentials, e.g., Martini force field, which was originally introduced for lipids^6^ and was later extended to other bioorganic and organic molecules.^7^ Other examples of coarse-grained models are the lipid model from the Deserno group,^8^ 3SPN DNA model,^9^ see also a recent review for others.^10^ A complicating circumstance in the formulation of a CG model is that (differently from atomistic models) even a functional form of the effective interaction potentials between CG sites is, in many cases, not known a priori, and traditionally used Lennard–Jones potentials may not be optimal for this purpose.

Within the multiscale bottom-up methodology, a CG force field is parameterized to fit some important physical properties obtained from a high-resolution (atomistic) simulation. Several bottom-up approaches to parametrize CG force fields have been formulated recently: force matching approach (also known as multiscale coarse-graining);^11−13^ reconstruction of effective potentials from radial distribution functions (RDFs) and other structural information (also called structure-based coarse-graining) by the inverse Monte Carlo (IMC)^14,15^ or iterative Boltzmann inversion (IBI);^16^ and relative entropy minimization.^17^ For example, the IMC method was used for computations of effective coarse-grained potentials in such complex macromolecular systems as lipid bilayers and assemblies,^15^ DNA,^18^ and nucleosome core particles.^19^ Furthermore, open-source software packages implementing structure-based coarse-graining with the inverse Boltzmann and inverse Monte Carlo methods have been developed.^20,21^

Although the area of bottom-up multiscale modeling shows fast progress and attracts much interest, a number of principal questions still remain unsolved. The transferability and representativity of the CG potentials are important issues since the CG potentials, in a general case, depend on the thermodynamic conditions of the atomistic simulations from which they were derived.^22^ Transferability of bottom-up CG models with respect to temperature and system composition (concentration) has been investigated in a number of works with respect to the force matching approach^23−25^ and IMC.^26,27^ Parameterization of CG models from atomistic simulations at different state points^24^ and inclusion of many-body interactions^28^ have been discussed as possible ways to improve the transferability of CG potentials. These approaches face, however, implementation difficulties in the traditional bottom-up determination of CG potentials.

Data-driven approaches, including machine learning and artificial neural networks (ANN or NN), have recently emerged as a novel way to formulate models for molecular simulations. In 2007, Behler and Parrinello^29^ suggested using the ANN framework to represent the energy surface of an atomistic system. Within this approach, the local arrangement of atoms is presented as a set of many-body descriptors, also called symmetry functions, since they are invariant to permutations of identical atoms as well as translations and rotations of the coordinate system. These descriptors are used as inputs to ANN, which is trained to fit the quantum–mechanical energy surface. Other systems of descriptors, such as Gaussian approximation potentials^30^ or smooth overlap of atomic positions (SOAPs),^31^ have been suggested. A well-trained ANN can be seen as a force field that includes many-body interactions and, for this reason, can reproduce an energy surface with nearly ab initio precision. Machine learning-derived atomistic force fields have been developed for a range of materials and proved their efficiency and robustness in a number of applications.^29,32,33^

An evident idea would be to use ML/ANN methods to develop coarse-grained force fields based on results of atomistic simulations, in the same manner as atomistic ANN force fields are trained with results of ab initio computations. A principal problem here is that in CG models, an analogue of a quantum–chemical surface is an N-body potential of mean force, which is not available from atomistic simulations. Recently, several attempts have been made to formulate ML methods for coarse-grained simulations based on the force matching approach by optimizing the average force acting on coarse-grained beads, which represent the gradient of the N-body potential of mean force.^34,35^ The method was used to build ANN potential for proteins to describe their dynamics.^36^ Also, the relative entropy minimization method has been applied to train ANN for CG models.^37^ An overview of some other approaches that use ML in coarse-graining is given in reviews.^38,39^ Furhermore, perspectives of coarse-grained ANN force fields are discussed in recent papers.^40,41^

Here, we propose an approach to build CG models combining ideas of the ML/ANN force field and structure-based coarse-graining using the IMC method. The idea is to train ANN on the reproduction of averaged structural information (e.g., RDF) obtained in atomistic simulations. We show that in the simplest case (linear ANN without intermediate layers and trained on a single atomistic simulation), the method is equivalent to the conventional IMC, while the inclusion of intermediate layers and training of ANN on multiple atomistic simulations carried out at different state points can provide more transferable CG models. As a proof of concept, we illustrate the approach on the Lennard–Jones fluid and then on a simple model of methanol–water mixtures, where the CG system is presented by single-site methanol molecules in implicit water.

## Methods

### Theoretical Background

#### Combining Machine Learning with Inverse Monte Carlo

**Figure 1:**
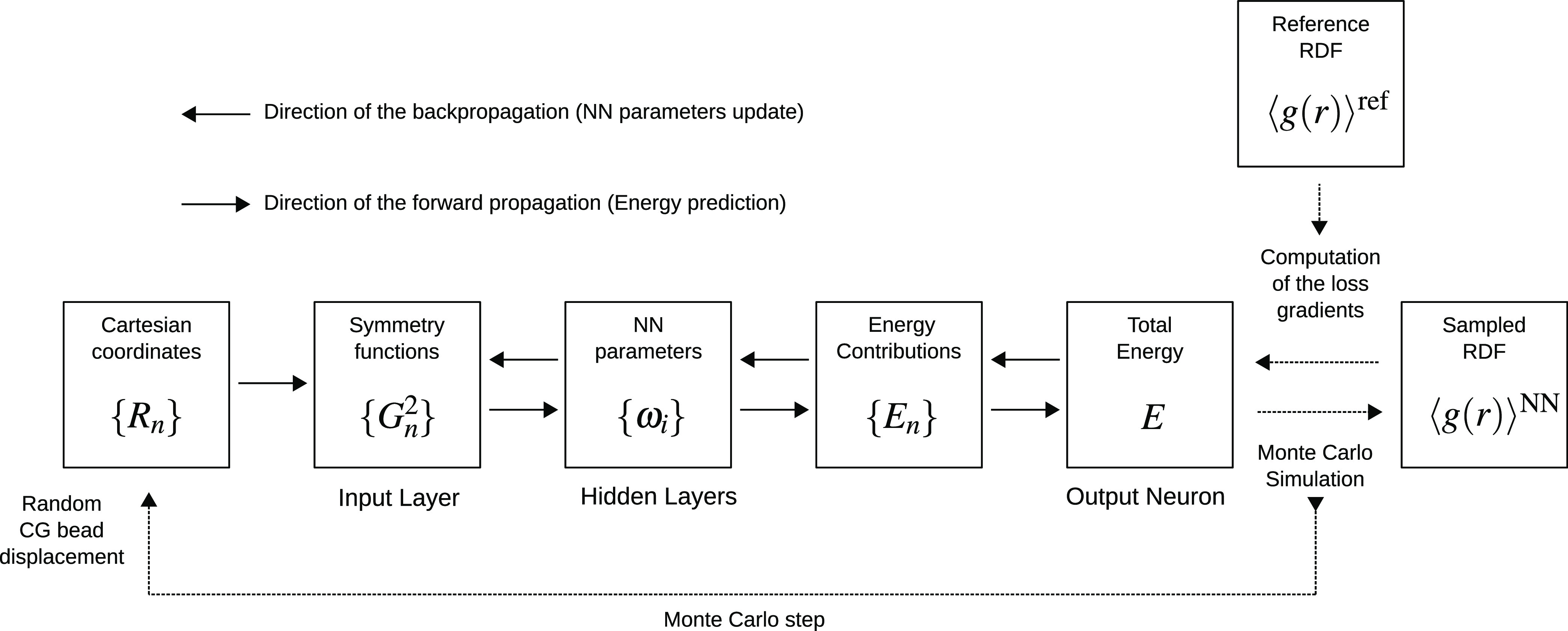
Training algorithm outline.

The proposed method uses the Behler–Parrinello neural network architecture^29^ to predict the total energy from a given configuration of interacting atoms. Furthermore, the training procedure utilizes ideas of the inverse Monte Carlo method^14^ by using statistical–mechanical relationships connecting change of interaction parameters with changes in RDF. The outline of the method is shown in Figure 1.

The method assumes that the system of interest (the reference system) is thoroughly sampled at atomistic resolution, and the resulting trajectory is mapped to the CG coordinates. Subsequently, both the RDFs for the CG sites and representative equilibrium system configurations at CG resolution are available. RDFs from several atomistic simulations carried out at different state points can be used for training ANN, but for simplicity, we describe here the case of training from a single atomistic simulation.

The training procedure consists of a series of Monte Carlo (MC) runs of the CG system with energy defined by Parrinello–Behler ANN in which the network parameters (weights) are optimized in order to provide the best possible fit of RDF to the reference one defined from the atomistic simulations. At each step of the MC procedure, the Cartesian coordinates of the current configuration are used to compute a set of radial symmetry functions (*G*^2^)^29^ for each CG site *n*, as shown in eq 1
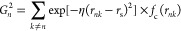
1

Here, the sum is taken over all other CG sites *k*, *r*_*nk*_ is the distance between sites *n* and *k*, parameter η controls the width of the Gaussian curve, and parameter *r*_s_ specifies the center of the Gaussian curve. The cutoff function *f*_c_(*r*_*nk*_), shown in eq 2, ensures a smooth decay of the *G*^2^ symmetry functions to zero at the cutoff distance *r*_c_
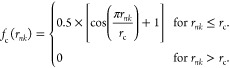
2

The resulting set of the radial symmetry functions for each CG site *n* {*G*_*n*_^2^} is used as the input data to the neural network with weight parameters {ω_*i*_} (no bias parameters are used), which returns the energy contribution *E*_*n*_ of the CG site *n*. The energy contributions of all CG sites in the system are summed into the total energy *E*
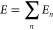
3

In the present work, only systems with one CG particle type are considered, and the neural networks used to predict the energy contribution for each CG site have the same parameters. We also do not use at present angular symmetry functions,^42^ noting that their inclusion into the algorithm is straightforward.

The procedure described above presents a way to compute the total energy for any given configuration of CG sites with ANN effectively acting as a force field. It is thus possible to use the neural network to sample RDFs by performing standard Metropolis Monte Carlo moves. After sufficient sampling, the RDF corresponding to the given neural network ⟨*g*(*r*)⟩^*NN*^ is compared to the known reference RDF ⟨*g*(*r*)⟩^ref^. The loss function of the ANN, *L*, is computed according to eq 4, where α is the index of the RDF bin
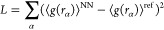
4

The next step is to compute the gradients of the loss function with respect to all NN parameters, ∂*L*/∂ω_*i*_, in a process known as backpropagation. The loss gradients are defined by eq 5
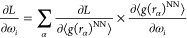
5

The first derivative in eq 5 follows directly from eq 4. The RDF, sampled with the ANN as the interaction potential, is not a direct ANN output; hence, it is not possible to compute the loss gradients following the standard backpropagation routines. However, the derivatives of statistical averages with respect to the potential parameters can be obtained with the help of statistical mechanics expressions in the same way as it is done in the inverse Monte Carlo method. Assuming no explicit relation between the statistical average ⟨*g*(*r*_α_)⟩ and potential parameters (ANN weights ω_*i*_), as well as substituting the Hamiltonian with the total energy provided by the neural network *E*, we get the following derivative of the RDF element with respect to ANN weights^43^
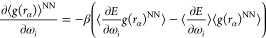
6where β = 1/*k*_B_*T*, *k*_B_ is the Boltzmann factor, *T* is the temperature in K, and *g*(*r*_α_) is the estimator of RDF for the sampled configuration. All of the terms within the averaging in eq 6 can be computed during the Monte Carlo sampling, and the energy derivative ∂*E*/∂ω_*i*_ is easily obtained, as *E* is an explicit function of the ANN parameters.

In order to improve the transferability of the CG ANN potential, training can be carried out on multiple atomistic simulations performed under different conditions. In this case, we run Monte Carlo sampling of multiple CG systems corresponding to each of the reference systems using the same neural network parameters (which can be done in parallel). The total loss function is defined as the sum of the loss functions of each of the systems. The resulting gradients of the loss function with respect to the NN parameters are used to update the neural network and minimize the total loss according to the optimization procedures. The Monte Carlo simulation is then repeated with the updated set of ANN weights to compute new RDFs and the loss function, and the iteration procedure is continued until convergence of the loss function is reached.

#### Pretraining: Improving the Initial Guess for the NN Parameters

To speed up the training process and reduce the number of training iterations for the convergence of the RDF loss function, we propose an efficient way for the initialization of NN parameters that we refer to as pretraining.

The algorithm is based on tuning the neural network parameters to reproduce the energy change during a Metropolis Monte Carlo step predicted with the potential of mean force (PMF) *V*(*r*), recovered from the RDF, as shown in eq 7
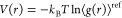
7

To avoid discontinuities in the core region where RDF is equal to zero, we substitute the PMF corresponding to zero RDF values with a smooth linear repulsive function, see eq 8
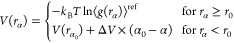
8where *r*_0_ is the shortest distance with nonzero RDF with index α_0_ and Δ*V* = *V*(*r*_α0_) – *V*(*r*_α0+1_). An example of such smooth PMF together with the corresponding RDF is shown for liquid argon at 95 K in Figure S1 of the Supporting Information.

The total energy predicted with PMF is computed as the sum of all pair interactions according to eq 9
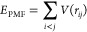
9

Since eq 7 provides (approximate) energy for each configuration, pretraining can be carried out similarly to the standard Behler–Parrinello procedure.^29^ We implemented pretraining in the following way. A random equilibrium configuration of CG sites is picked by using one of the snapshots of the reference trajectory. Then, the total energy of the configuration is predicted by both the neural network (initially starting with random parameters from −1 to +1) and PMF. In the next step, a single CG bead is randomly displaced, and the energy is evaluated again with both the NN and PMF. A loss function is constructed from the squared difference of the predicted energy changes by NN and PMF, as given in eq 10
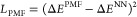
10

The loss gradients with respect to the NN parameters are computed according to eq 11
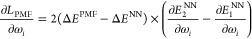
11where *E*_2_^NN^ is the total energy of the configuration after the displacement and *E*_1_^NN^ is the total energy of the original configuration, both predicted by the NN.

The computed gradients are then used to update the NN parameters. Similarly to the main part of the training, the gradients can be computed with several reference systems and subsequently averaged. The next pretraining step is done by picking another random equilibrated configuration, performing a random displacement, and recomputing the loss gradients. The pretraining steps are much quicker to perform than the main training iterations, and after running sufficiently many pretraining steps, the neural network provides a significantly better guess for the interaction potential compared to the random initialization. The pretrained neural network can be used as a starting point for the main training for the further refinement of the interaction potential.

### Reference Atomistic and IMC Simulations

We tested the method on two systems. The first one is a system of particles interacting by a Lennard–Jones potential corresponding to liquid argon, which is done with the purpose of reproducing a system interacting by a known potential. The second system is a single-site coarse-grained model of methanol in a water–methanol mixture that is trained by atomistic simulations carried out at different methanol concentrations. A similar single-site CG methanol model has been previously used to investigate the transferability of force matching-derived interactions with respect to temperature^25^ and the presence of surfaces.^28^

#### Liquid Argon System

The reference data for the liquid argon system (Lennard–Jones system) are obtained with a Metropolis Monte Carlo sampling of 512 Ar atoms in a 3D periodic simulation box using a Monte Carlo engine, which is also used to train the models. The simulation is run at *T* = 95 K and ρ = 1374 kg/m^3^ using the Lennard–Jones parameters from the work of Rowley et al.^44^ for a total of 1.2 billion MC steps with 60 million steps for equilibration and an output frequency of 1 million steps^–1^. The Ar–Ar RDF is computed with a distance range from 0 to 10 Å and 400 total RDF bins (bin width of 0.025 Å). The target acceptance ratio is set to 0.5. A total of 200 frames are picked from the Monte Carlo trajectory to serve as starting points for the training.

#### Water–Methanol Atomistic Simulation Setup

The reference data for the water–methanol systems were obtained with atomistic molecular dynamics (MD) simulations using the general Amber force field (GAFF)^45,46^ for methanol and the TIP3P water model.^47^ The MD simulations were carried out using GROMACS 2020.4 software.^48−53^ The MD simulation setup is described as follows: 512 methanol molecules are placed in a 3D periodic cubic simulation box with the box size estimated from the expected density of the desirable methanol concentration, and the water molecules are added to the simulation box according to Table 1, giving a total of 10 methanol–water systems with the molar concentration of methanol ranging from 10 to 100 mol %. The energy of each simulation box is minimized using the steepest descent algorithm, and a 1 ns long equilibration with the Bussi thermostat^54^ (*T* = 298.15 K, τ_T_ = 0.1 ps) and the Berendsen barostat^55^ (*p* = 1 atm, τ_p_ = 2.0 ps) is carried out. The production simulation length ranges from 100 ns for pure methanol to 1000 ns for 10 mol % methanol. The Parrinello–Rahman barostat^56^ (*p* = 1 atm, τ_p_ = 5.0 ps) is used to control the pressure. A time step of 2 fs is used for all MD runs. The cutoff radius for both VdW and short-ranged electrostatic interactions is set to 1 nm. The Verlet cutoff scheme^57^ with the buffer tolerance of 0.005 kJ × mol^–1^ × ps^–1^ per atom is used to generate the pair lists. Long-range electrostatics are computed with the particle mesh Ewald (PME) method^58^ with a grid spacing of 0.12 nm and cubic interpolation. Long-range corrections to both the energy and pressure are performed for VdW interactions. All bonds with hydrogen atoms are constrained using the LINCS algorithm.^59^ The output frequency is set to 5 ps^–1^. Visualization of the simulations is done with VMD.^60^

**Table 1:** Methanol–Water Systems in Atomistic MD Simulations

N(CH_3_OH)	N(H_2_O)	approx. CH_3_OH conc., mol %	box size, Å	density, kg/m^3^
512	0	100%	32.3	808.2
512	57	90%	32.8	819.5
512	128	80%	33.4	830.7
512	220	70%	34.2	843.3
512	340	60%	35.2	856.4
512	512	50%	36.5	871.2
512	768	40%	38.4	887.7
512	1195	30%	41.1	906.6
512	2048	20%	45.7	928.7
512	4608	10%	55.6	954.6

#### CG Mapping and Reference RDF Preparation

**Figure 2:**
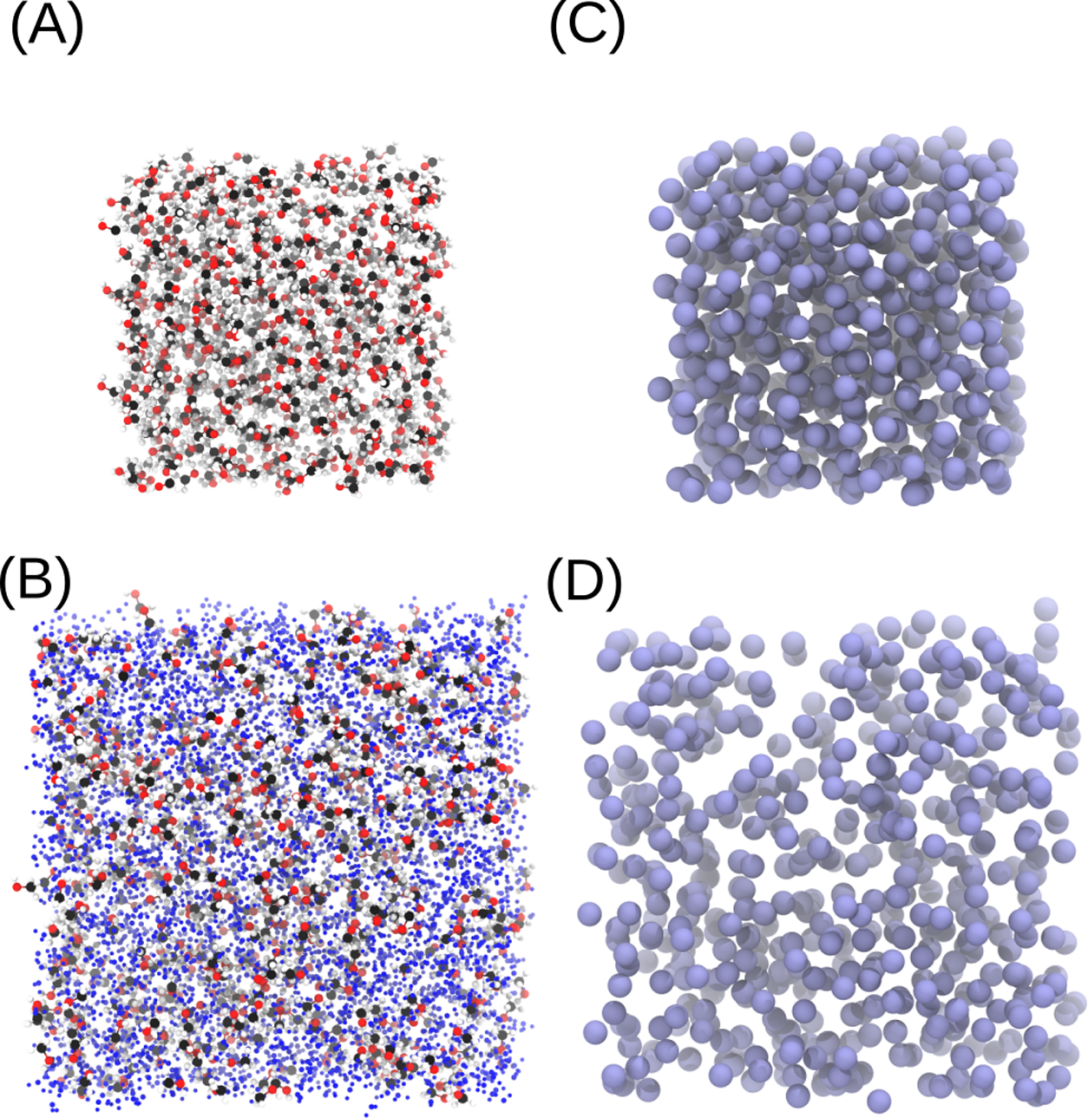
Atomistic and coarse-grained representations of methanol–water mixtures. Pure methanol at the atomistic resolution (A) and the coarse-grained resolution (C) and the methanol–water mixture (20 mol % CH_3_OH) at the atomistic resolution (B) and the coarse-grained resolution (D).

The resulting atomistic trajectories are mapped to CG coordinates in which a methanol molecule is represented by a single CG site located in the molecular center of mass, and the water is completely excluded from the CG representation. The coarse-grained mapping is performed by the cgtraj utility from the MagiC 3 software package.^21^ The RDF for each methanol system is computed by using the production part of the trajectory with a distance range from 0 to 15 Å and 300 total RDF bins (bin width of 0.05 Å). A total of 200 frames are picked from each trajectory to serve as starting points for the coarse-grained simulations. Snapshots of atomistic and coarse-grained representations of pure methanol and methanol–water solutions are shown in Figure 2.

#### Reference Inverse Monte Carlo Optimization

In order to compare the proposed method with coarse-graining using the inverse Monte Carlo method, we used five different methanol–water simulations (20, 40, 60, 80, and 100 mol % methanol) to compute five corresponding CG effective potentials using the IMC method. For each system, we run 10 IMC iterations with a total of 50 million Monte Carlo steps (half being used to equilibrate the system) per CPU core. The first five iterations are run on 128 CPU cores with the regularization parameter set to 0.5. The last five iterations are run on 256 CPU cores, and the regularization parameter is set to 1.0. The IMC simulations are carried out at the same temperature as the atomistic MD simulation (*T* = 298.15 K) and at a constant volume corresponding to the average box size obtained in the atomistic simulations (see Table 1). The target acceptance ratio is set to 0.5, the output frequency is set to 1000 steps^–1^, the cutoff radius is set to 15 Å, and the RDF is computed with 0.01 Å resolution. The IMC simulations are carried out by the MagiC 3 software package.^21^

### Training the Coarse-Grained ANN Potentials

#### Software

We have implemented the training method using the Julia programming language (version 1.8)^61^ with the Flux machine learning library,^62,63^Zygote^64^ library for automatic differentiation, and Chemfiles software^65^ for handling reading and writing trajectory files. Additionally, the software uses the Distributed Julia package to run the Monte Carlo sampling on multiple CPU cores.

#### General Training Procedure

The general training procedure is described as follows. After the atomistic simulations are carried out, the original coordinates are mapped to the CG coordinates, the RDF is computed, and a number of equilibrium configurations of CG sites are picked for later use as starting configurations for the training. Then, an appropriate set of symmetry functions must be selected. One has to decide on the number of symmetry functions per CG bead and the corresponding symmetry function parameters. For *G*^2^ symmetry functions, they include the η parameter (controls the Gaussian curve width), the *r*_s_ parameter (center of the Gaussian curve), and the cutoff radius *r*_c_. The next step is to select the NN hyperparameters, which are the number of hidden layers and neurons in each layer, activation functions, learning rates, and the optimizer algorithm. After all of the input parameters are set, the ANN weights are first optimized during the pretraining procedure and refined later during the main part of the training.

#### Selected Hyperparameters

We have made test training computations varying the number of *G*^2^ functions and their parameters, the number of intermediate network layers, and the number of neurons in them. These computations showed that the *G*^2^ symmetry function parameters have the greatest influence on the training process. An illustration of the effect of the number of *G*^2^ symmetry functions and the corresponding η and *r*_*s*_ parameters for training the CG methanol–water system on RDFs obtained in four atomistic simulations at 10, 40, 60, and 100% concentrations is shown in Figure S2 of the Supporting Information. We found that the presence of a wide *G*^2^ function centered at zero greatly improves the convergence of the RDF in the core region, and we have used such a symmetry function in all computations. Furthermore, while choosing between sets of *G*^2^ functions that provide nearly equal quality results, we give preference to the one with the minimal number of *G*^2^ functions since the computation of *G*^2^ functions is the most time-consuming part of the training process. So, finally, we selected two different sets: one containing 8 *G*^2^ functions (Set 1) and the second one containing 24 *G*^2^ functions (Set 2). We name the two corresponding models as Model 1 and Model 2, respectively. Both sets have a wide *G*^2^ function centered at 0 Å with η = 0.125 Å^–2^, and seven others have their centers uniformly distributed in the 3–6 Å range with a step of 0.5 Å and η = 4.0 Å^–2^. The second set used in Model 2 additionally uses a wider *G*^2^ function centered at 0 Å with η = 0.02 Å^–2^, nine narrower functions with η = 16.0 Å^–2^ between 3 and 5 Å, and finally, five more functions in the 3–8 Å range and η = 2.0 Å^–2^. The full list of *G*^2^ function parameters used in the production and test calculations is presented in Table S1.

We have further chosen the rectified linear unit (ReLU), shown in eq 12, to introduce nonlinearities in our neural networks and the Adam-derived optimizer algorithm called AMSGrad.^66^ It has been shown that storing more information about past gradients fixes some of the convergence issues that the original Adam optimizer has.
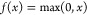
12

The effect of the number of hidden layers and the neurons in each layer on the training process is shown in Figure S3 of the Supporting Information. We have finally selected three additional hidden layers for both models, with 20 neurons in each for Model 1 and 40 neurons in each for Model 2.

## Results

### Model Training

#### Lennard–Jones Potential

First, we test the method on a simple system interacting with the Lennard–Jones pair potential representing liquid argon. We show that the proposed method is capable of training a simple linear neural network consisting of only eight input neurons, each transferring the value of the respective *G*^2^ function to the output layer consisting of one neuron, giving the energy value through a linear activation function. This energy is used to sample the system using a standard MC algorithm and compute the RDF, which is further used to optimize the network weights. For this case, we use the smallest set of *G*^2^ functions (Set 1) shown in Table S1. The total energy, predicted by this linear neural network and the symmetry functions, is expressed by eq 13
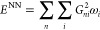
13where *n* is the CG site index and *i* is the *G*^2^ or the weight index.

The equation for the total energy computed with the linear neural network is, in fact, very similar to the expression of the total energy of a coarse-grained system according to the IMC method, where, instead of the symmetry functions, a histogram of the pair distances between the particles is used. *G*^2^ symmetry functions are approaching values of the histogram of pair distances at sufficiently small widths of the Gaussians, regularly separated *r*_s_ parameters, and large cutoff. If one uses a histogram of pair distances as input parameters of such a linear network, eq 6 becomes identical to the one used within the IMC method, with weights ω_*i*_ having the sense of pair potential in the corresponding bin of the histogram. Noteworthy, this example shows that even a simple linear network allows reducing the number of input parameters to less than ten, while the histogram of pair distances in the IMC method typically uses hundreds of bins.

**Figure 3:**
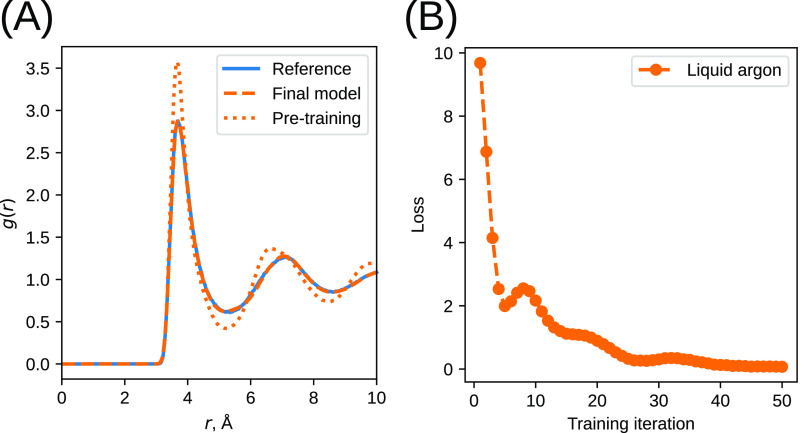
(A) Comparison of the reference RDF and the RDF sampled with NN for liquid argon at 95 K and (B) loss convergence for the liquid argon at 95 K.

For the LJ system, we started the optimization process with 50 000 pretraining steps to improve our initial guess of the ω_*i*_ parameters. After the pretraining, a total of 50 training iterations followed. Each iteration runs a total of 8 million Monte Carlo steps with 2 million for equilibration. The final RDF is shown in Figure 3(a), and the loss convergence plot is shown in Figure 3(b).

One can observe that in this case, the pretraining provides a reasonable starting guess for the NN, and the main part of the training further improves the model until the RDF, sampled with the NN as the potential perfectly coincides with the reference RDF. The loss decreased significantly from 9.7 to 2.0 during the first five iterations after the pretraining, and then it decreased to 0.07, providing perfect agreement with the reference RDF.

#### Water–Methanol CG Model: A Linear Model

**Figure 4:**
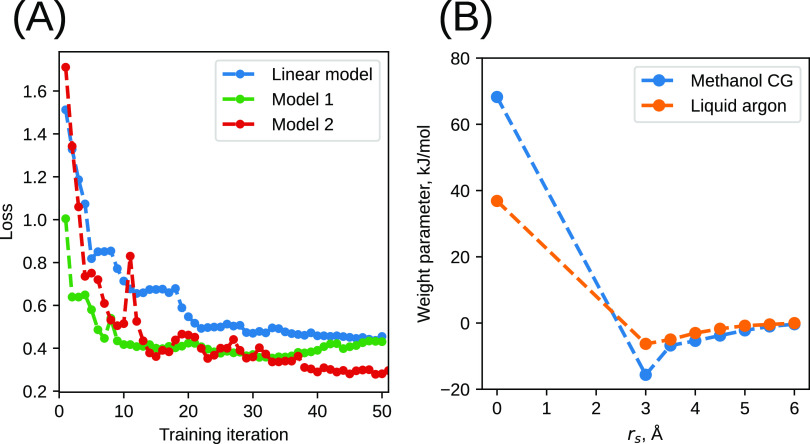
(A) Convergence of the loss function for the CG methanol models. (B) Optimized linear NN weights for the methanol–water system (blue) and for the liquid argon system (orange), as a function of *G*^2^ symmetry function *r*_s_ parameter.

In the next test case, we train a linear model to reproduce the structure of CG methanol dissolved in implicit water using RDFs from several atomistic simulations carried out at 10, 40, 60, and 100 mol % methanol as a reference. First, we use the same NN architecture (only eight weight parameters with linear activation) and the same set of *G*^2^ symmetry functions with all η, *r*_s_, and *r*_c_ parameters as in the Lennard–Jones case (Set 1). Here, we start with 50 000 pretraining iterations and continue with 50 main training iterations. Each main training iteration consists of 8 million Monte Carlo steps per reference system. The decrease in the mean loss function (averaged over the loss value for each methanol concentration) is shown in Figure 4A. As in the liquid argon case, the loss decreases sharply at the beginning of the training and levels out later, after around iteration 25. It is noteworthy that the optimization process appears to approach a loss value that is noticeably larger than what was observed in the Lennard–Jones system. This can be expected because we are trying to fit RDFs of several atomistic simulations carried out at different state points within the same ANN, and the model based on only *G*^2^ symmetry functions may not be flexible enough to accommodate the structural differences of methanol–water solutions at different concentrations.

As we discussed in the previous section, in the case of linear networks, the NN weights are related to the effective pairwise potential at the distances corresponding to Gaussian centers of the *G*^2^ parameters. Figure 4B shows the weights of the optimized linear NN both for the CG methanol model and discussed in the previous section of the Lennard–Jones system. One can see that the weights of the two models have some similarities between each other and also with the Lennard–Jones-like interaction potential, with a high positive value at zero, the lowest value at a typical contact distance of 3 Å, and approaching zero at larger distances.

#### Water–Methanol CG Potential: Nonlinear Models

Finally, we have trained more complex neural networks that also take symmetry functions in the input layer but incorporate intermediate layers and ReLU activation function to introduce nonlinear behavior. Two such models are used in the production: Model 1 with 8 *G*^2^ functions as in the case of Lennard–Jones fluid and the linear methanol CG model (see Set 1 in Table S1) and Model 2 that takes 24 *G*^2^ functions as the input data (see Set 2 in Table S1). The same reference systems as in the linear methanol CG model are used for both models. As in the other examples, we begin with 50 000 pretraining steps and then continue with main training iterations. The mean loss as a function of the number of iterations is shown in Figure 4A. It is seen that both nonlinear models are able to reach lower loss values (both Model 1 and Model 2 reach a loss value of around 0.38 at iteration 25, while the linear model reaches only 0.51). Further training of Model 1, however, does not decrease the loss; hence, the state of Model 1 at iteration 25 is used in the production runs. Model 2, on the other hand, continues the downward trend and reaches a loss value of 0.27 after 50 iterations. The heatmaps of the optimized model parameters for both Model 1 and 2 are shown in Figure S4 of the Supporting Information.

### Model Validation

#### Validation Simulation Setup

The quality of the resulting models is assessed by running Monte Carlo simulations, computing RDFs with fixed neural network parameters, and comparing the results with the reference RDFs. Furthermore, we compute the angle distributions for all of the triplets within the first coordination sphere of methanol molecules (up to 6.2 Å). It is important to note that, unlike the radial distribution functions, the neural networks were not trained on the angle distributions; thus, their comparison provides an independent assessment of the quality of the models. For CG methanol–water systems, the optimized neural networks are tested by comparison with atomistic results carried out at all-available methanol concentrations (from 10 to 100 mol %), including those that were not used during the training. Each Monte Carlo validation simulation was run for a total of 40 million Monte Carlo steps, with 4 million steps reserved for equilibration, at the same temperature (*T* = 298.15 K) as the atomistic MD simulation and at the constant volume. To further assess our ANN models, we perform similar validation simulations on all methanol systems using the CG methanol effective potentials obtained with IMC. All of the Monte Carlo simulations with IMC potentials are run for 50 million steps with 1 million steps for equilibration at the same conditions as respective ANN MC simulations.

#### Validation of Water–Methanol CG ANN Potentials

**Figure 5:**
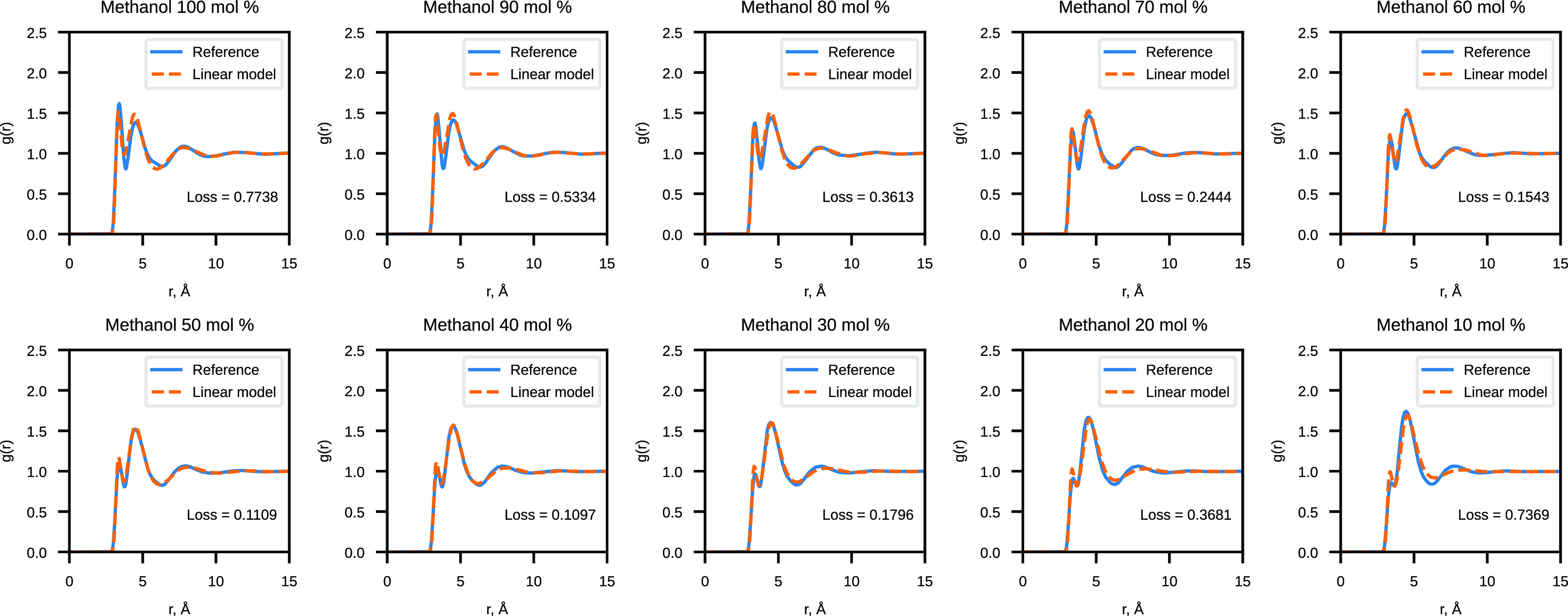
Comparison of the reference RDF and the RDF sampled with the linear model for CG water–methanol mixtures.

**Figure 6:**
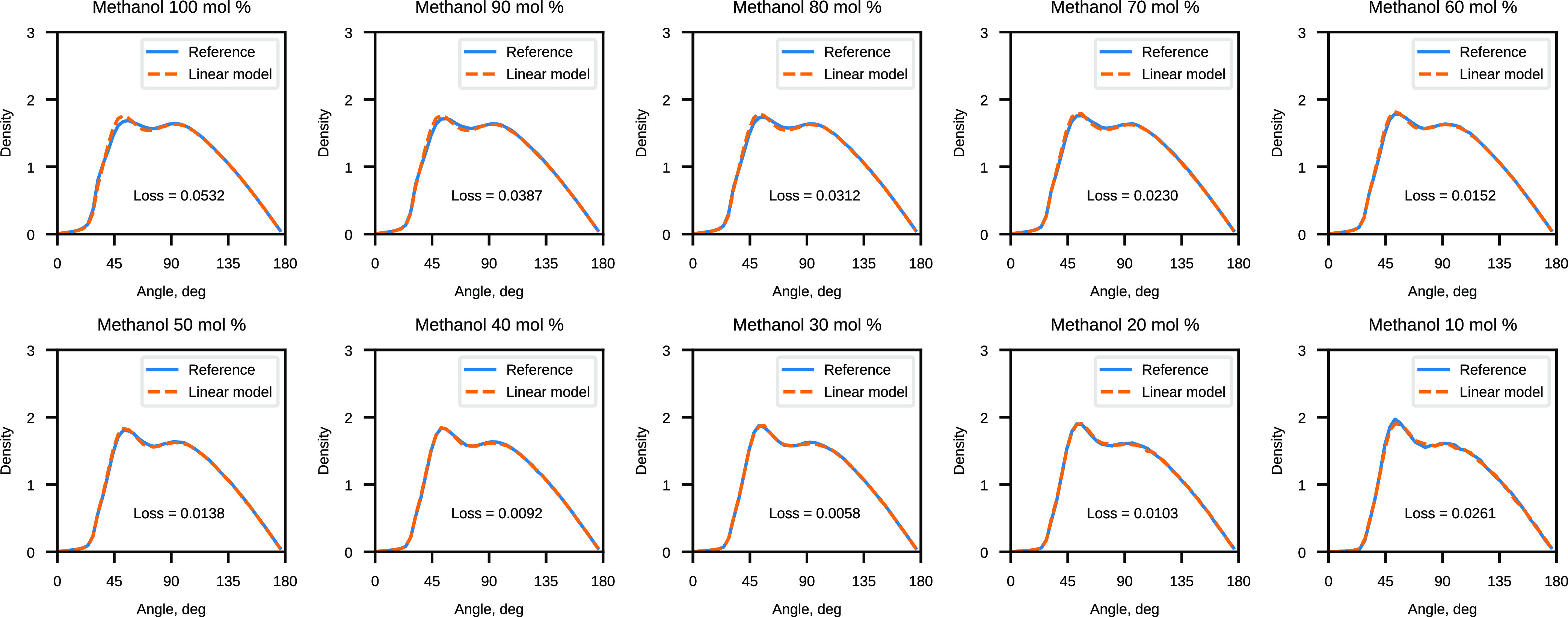
Comparison of the reference angular distribution and the distribution sampled with the linear model for CG water–methanol mixtures.

The results of the CG methanol linear model validation are shown in Figures 5 and 6. All computed angular distributions are normalized to probability densities, which are subsequently multiplied by the number of bins to make the new density values independent of the way in which they were partitioned. The model performs the worst at the “edge” concentrations of 10 and 100 mol %, but the prediction improves for the systems in the middle of the concentration range. It is possible that the higher weight of average concentrations (40 and 60 mol %) in the training systems might be the cause of the better prediction for those water–methanol systems.

**Figure 7:**
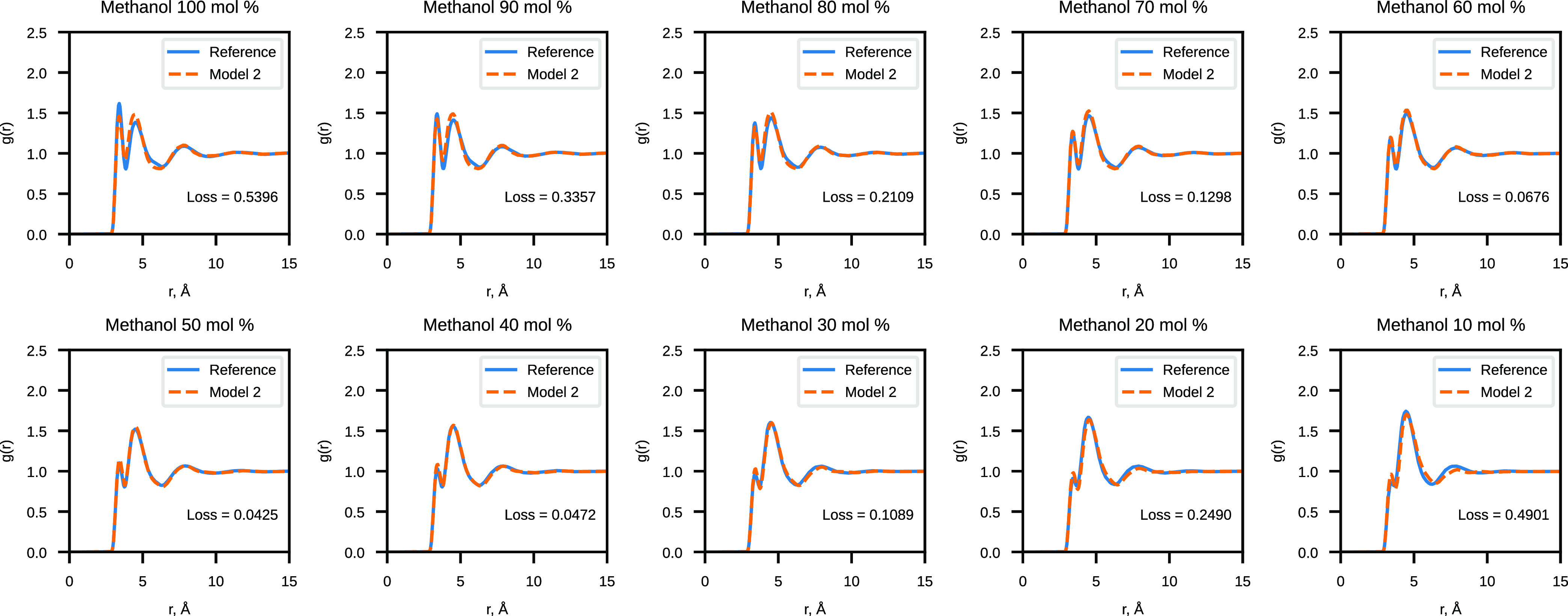
Comparison of the reference RDF and the RDF sampled with Model 2 for CG water–methanol mixtures.

**Figure 8:**
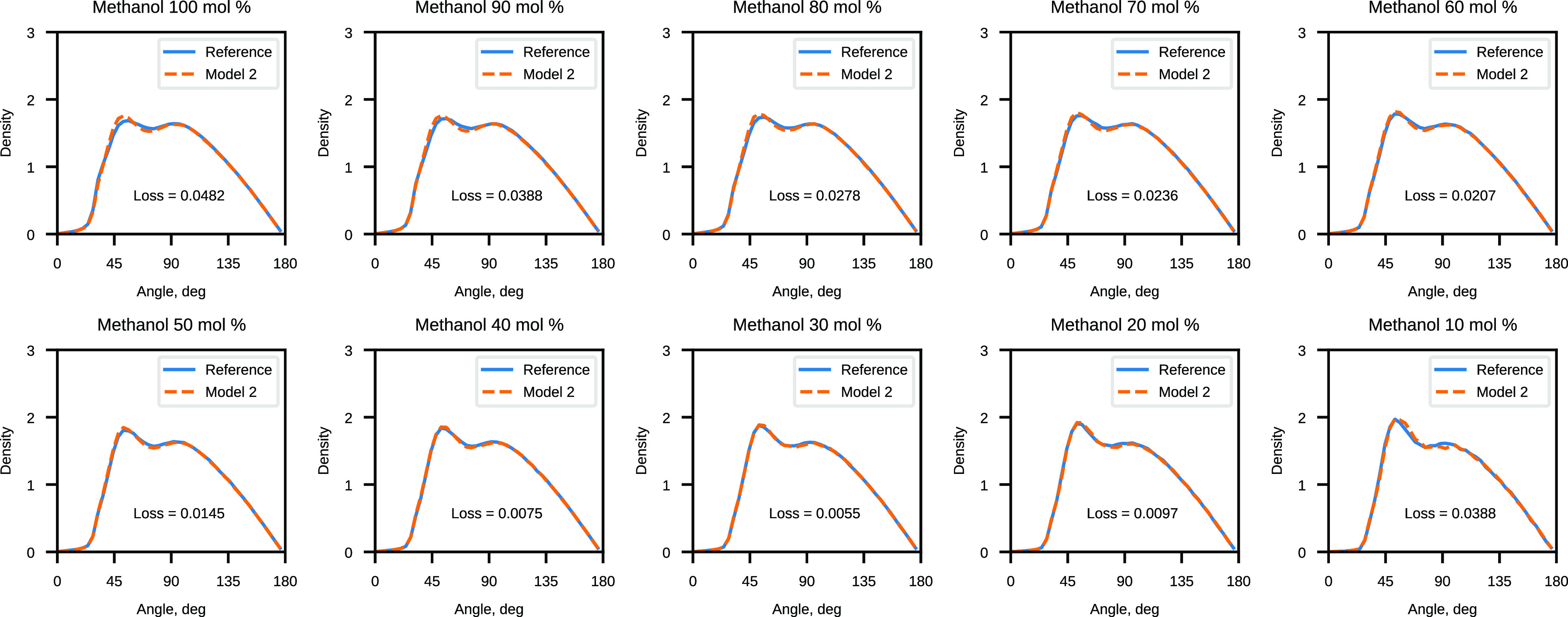
Comparison of the reference angular distribution and the distribution sampled with Model 2 for CG water–methanol mixtures.

The results of the validation of Model 2 are presented in Figures 7 and 8. Model 1 validation results can be found in the Supporting Information (Figures S5 and S6). We observe a significant reduction of the RDF loss across the whole range of concentrations when comparing Model 2 results to the linear model with eight *G*^2^ functions. Model 1 similarly provides lower RDF loss values than the linear model but not as dramatically lower as Model 2. Angular distributions are well reproduced by both nonlinear models and the linear model.

#### Comparison with the IMC Simulations

**Figure 9:**
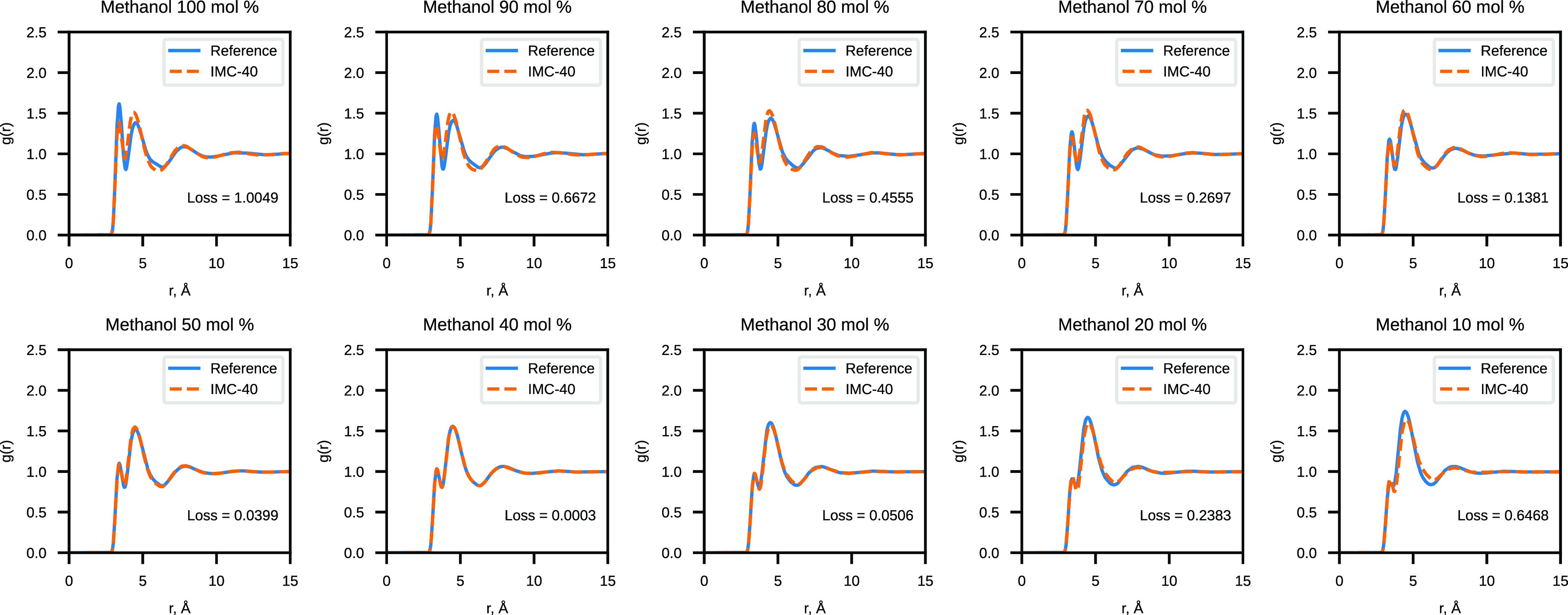
Comparison of the reference RDF and the RDF sampled with the IMC effective potential trained on 40 mol % methanol.

Comparing the obtained NN models with the IMC effective potentials may provide further insight into the transferability of the CG potentials. Figures 9 and S7 of the Supporting Information show how the IMC effective potential trained with a 40 mol % methanol system has performed when simulating all of the reference water–methanol systems. This is the best-performing IMC potential, and the results of the other IMC potentials are shown in Figures S8–S15.

**Figure 10:**
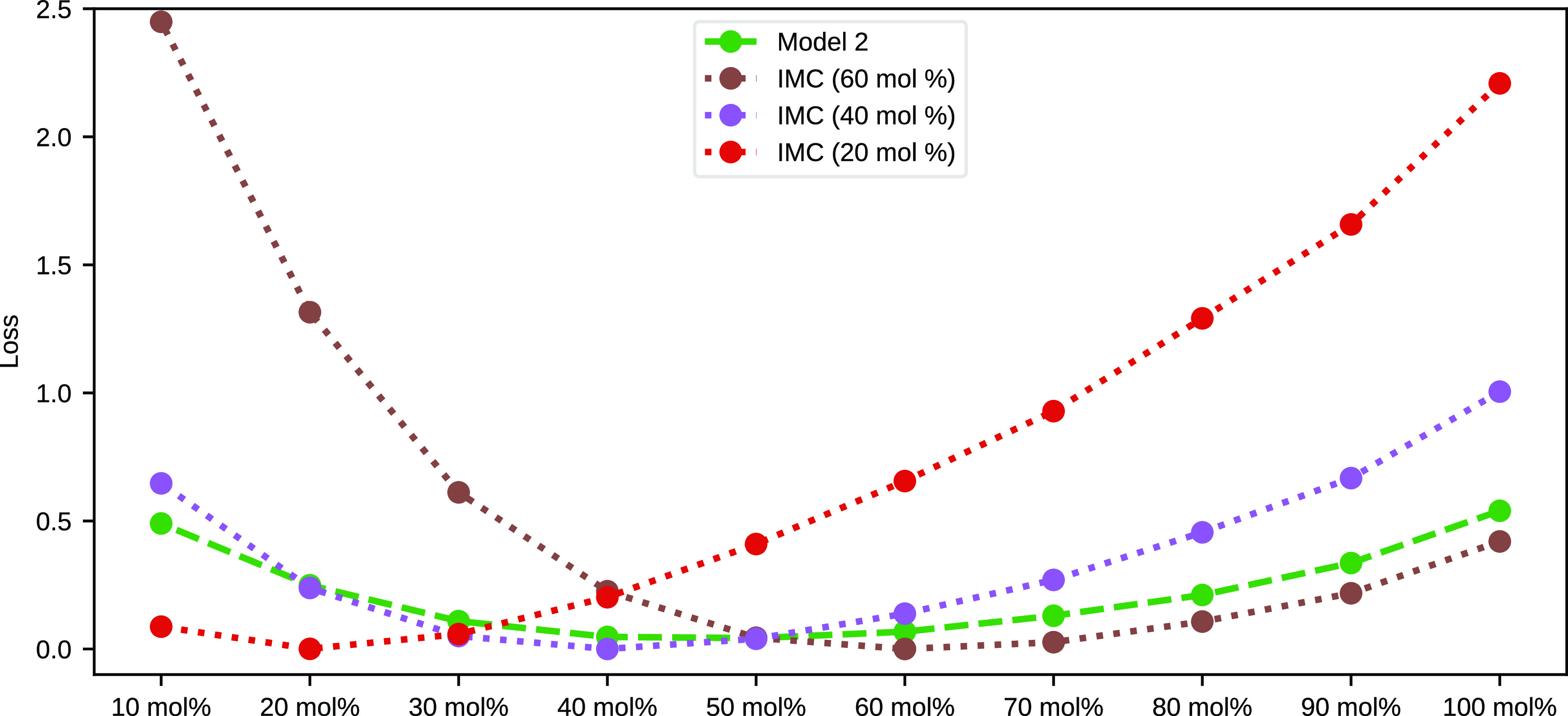
Comparison of the radial distribution function loss values obtained for Model 2 and three of the best IMC models.

The summary of the loss data where the linear and nonlinear models are compared with the IMC models is presented in Tables 2 and 3. Also, Figures 10 and S16 show the loss values calculated for each methanol–water system obtained with Model 2 and some of the best-performing IMC effective potentials.

**Table 2:** Comparison of the Radial Distribution Function Losses from Different Methanol CG Models^a^

	linear	model 1	model 2	IMC-100	IMC-80	IMC-60	IMC-40	IMC-20
100 mol %	0.7738	0.6253	0.5396	0.0002	0.0997	0.4200	1.0049	2.2086
90 mol %	0.5334	0.4179	0.3357	0.0603	0.0347	0.2176	0.6672	1.6578
80 mol %	0.3613	0.2860	0.2109	0.0925	0.0001	0.1071	0.4555	1.2910
70 mol %	0.2444	0.1947	0.1298	0.2175	0.0270	0.0266	0.2697	0.9288
60 mol %	0.1543	0.1315	0.0676	0.3996	0.1038	0.0002	0.1381	0.6557
50 mol %	0.1109	0.1110	0.0425	0.7553	0.2909	0.0438	0.0399	0.4097
40 mol %	0.1097	0.1295	0.0472	1.5551	0.7374	0.2255	0.0003	0.2022
30 mol %	0.1769	0.2007	0.1089	3.1430	1.5926	0.6116	0.0506	0.0582
20 mol %	0.3681	0.3638	0.2490	6.2870	3.1816	1.3150	0.2383	0.0006
10 mol %	0.7369	0.6429	0.4901	11.7863	5.7367	2.4490	0.6468	0.0872
**mean loss**	0.3572	0.3103	0.2221	2.4295	1.1805	0.5416	0.3511	0.7500
**min loss**	0.1097	0.1110	0.0425	0.0002	0.0001	0.0002	0.0003	0.0006
**max loss**	0.7738	0.6429	0.5396	11.7863	5.7367	2.4490	1.0049	2.2086

a IMC-X is effective potential trained on a methanol–water system with X mol % methanol.

**Table 3:** Comparison of the Angular Distribution Losses from Different Methanol CG Models^a^

	linear	model 1	model 2	IMC-100	IMC-80	IMC-60	IMC-40	IMC-20
100 mol %	0.0532	0.0382	0.0482	0.0040	0.0117	0.0333	0.0673	0.1367
90 mol %	0.0387	0.0328	0.0388	0.0104	0.0120	0.0192	0.0500	0.1056
80 mol %	0.0312	0.0222	0.0278	0.0065	0.0040	0.0157	0.0352	0.1034
70 mol %	0.0230	0.0221	0.0236	0.0169	0.0052	0.0062	0.0311	0.0708
60 mol %	0.0152	0.0185	0.0207	0.0298	0.0096	0.0038	0.0172	0.0544
50 mol %	0.0138	0.0134	0.0145	0.0665	0.0196	0.0023	0.0121	0.0389
40 mol %	0.0092	0.0054	0.0075	0.1619	0.0612	0.0160	0.0037	0.0224
30 mol %	0.0058	0.0086	0.0055	0.3617	0.1600	0.0482	0.0062	0.0112
20 mol %	0.0103	0.0135	0.0097	0.7934	0.2533	0.0855	0.0119	0.0054
10 mol %	0.0261	0.0257	0.0388	1.2850	0.5930	0.1864	0.0381	0.0163
**mean loss**	0.0227	0.0200	0.0235	0.2736	0.1130	0.0417	0.0273	0.0565
**min loss**	0.0058	0.0054	0.0055	0.0040	0.0040	0.0023	0.0037	0.0054
**max loss**	0.0532	0.0382	0.0482	1.2850	0.5930	0.1864	0.0673	0.1367

a IMC-X is the effective potential trained on a methanol–water system with X mol % methanol.

## Discussion

The results presented in the previous section illustrate the ability of the NN models to reproduce the CG methanol structure over a wide range of concentrations with a lower mean loss than the IMC models. Even though the IMC models provide an excellent agreement for the systems on which they are trained, the loss increases significantly for the systems that are far from the reference concentration, and the maximum loss across the whole range of methanol–water systems is lower for all NN models compared even to the best-performing IMC model. Nonlinear multilayer NNs provide lower mean RDF loss and more uniform coverage of the whole concentration range.

**Figure 11:**
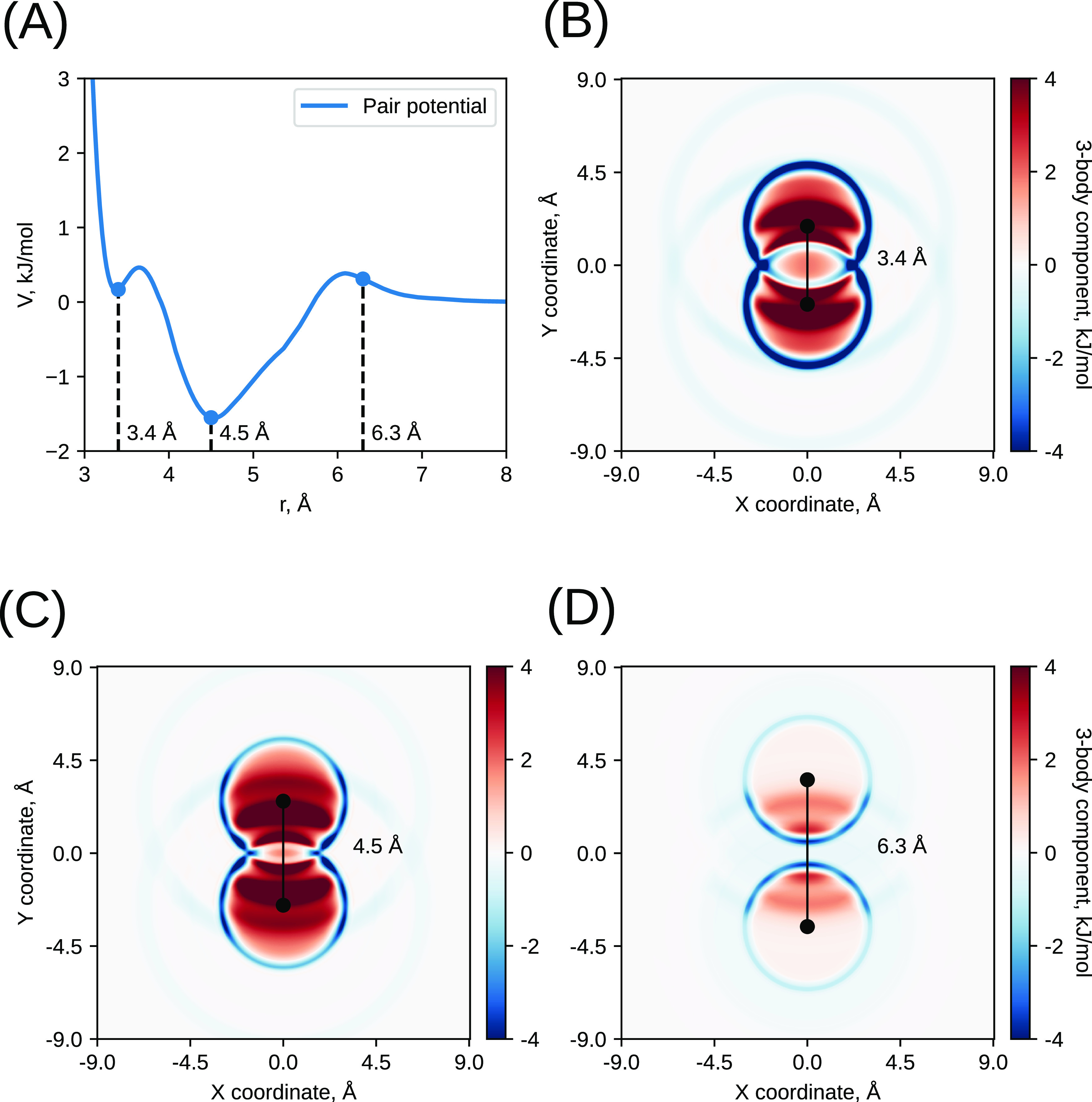
Illustration of the three-body component of the ANN potential trained for the nonlinear CG methanol model (Model 1). (A) Pair component of the ANN potential computed as the energy of two CG particles and (B–D) three-body component of the ANN potential computed as the energy difference Δ*E*^(3)^ = *E*^(3)^(1, 2, 3) – *E*^(2)^(1, 2) – *E*^(2)^(2, 3) – *E*^(2)^(1, 2), where *E*^(3)^(1, 2, 3) is the energy of three CG particles and *E*^(2)^(*i*, *j*) is the energy of each of the three particle pairs. The three-body component is shown as a density map of the position of the third particle when the first two particles are fixed at distances of 3.4, 4.5, and 6.3 Å, respectively.

It is important to note that for the considered methanol–water example in this work, the ANN models provide correct (and better than in the IMC approach) representation of three-body angular distributions, which were not used during the training process. In fact, the energy of neural networks built on only *G*^2^ symmetry functions includes a three-body interaction, as illustrated in Figure 11, that shows the difference between the ANN-computed energies of three particles and energies of pair interactions of these particles using Model 1. While *G*^2^ functions are computed from pair distances only, the many-body interaction appears in the total energy due to the following two reasons: (i) *G*^2^ functions are computed for each atom separately and not averaged over atoms, and (ii) each *G*^2^ function collects information over the range of distances determined by the shape of Gaussian. As we noted in the section “Selected hyperparameters,” one of the important symmetry functions that we used in all considered examples is the one centered at zero distance and having a wide shape. This symmetry function counts a number of particles in close vicinity of the given particle determined by the width of the Gaussian, and the associated NN weights work effectively as a “local density potential” previously considered as a way to improve the accuracy and transferability of CG models based on pair potentials.^28^

The most pronounced feature of the three-body interaction shown in Figure 11 is an enhancement of attraction at the contact distance (blue rings at about 3.4 Å distance corresponding to the first RDF maximum), which, at closer inspection, is seen as angularly dependent. This interaction appears when ANN is trying to fit RDFs for different concentrations and particularly to reproduce the increase of the first RDF peak (corresponding to methanol–methanol interactions through OH groups) at higher methanol concentrations. The IMC model based on pair potentials reproduces the balance between the first and second RDF peaks for different methanol concentrations substantially less accurately than the ANN models. The multibody character of ANN interaction thus improves the transferability of the model and provides a correct description of three-body angular distributions.

We note further that the transferability of the model with respect to solute concentration can be more important in CG simulations of nonhomogeneous systems under conditions of phase separation or aggregation. For example, one of the problems of CG modeling of self-assembly of lipids into aggregated structures is that effective pair interactions between the lipids depend on their concentration,^27^ and it is different, for example, for lipids dissolved in a solution and gathered in a bilayer. ANN potentials, transferable with respect to concentration, can bring substantial improvement into the modeling of such systems.

We hypothesize that the agreement between ANN-based CG and atomistic models may be further improved with better tuning of the network hyperparameters and the *G*^2^ symmetry function parameters. Also, the maximum loss can be likely reduced by introducing a nonuniform weighting of the gradients when updating the NN parameters. It is also likely that further improvement of the transferability of the ANN-based CG model can be reached by introducing three-body *G*^3^ symmetry functions as descriptors in the first network layer.

## Conclusions

In this paper, we introduced a method to train an artificial neural network to determine energy for a coarse-grained model using structural data obtained in atomistic simulations. The method has similarities with the inverse Monte Carlo in the sense that it uses structural information presented by RDFs to train the model; however, differently from IMC, it, in a natural way, can include multiple atomistic simulations carried out at different state points and thus provide a substantial improvement of the transferability of the model. We have tested the method on two simple systems, one representing liquid argon described by a Lennard–Jones potential and the other representing a single-site CG model of methanol in implicit water. In the latter case, multiple reference atomistic simulations carried out at different methanol concentrations were used, which allowed us to train the ANN with substantially better transferability compared to the IMC-derived CG model.

Concerning the computational expenses of the proposed method, we notice that in both considered examples, the number of training iterations and the number of MC steps per iteration are similar to the IMC method. The time for a single MC step depends on the number and type of input descriptors but is less dependent on the number of intermediate neurons and layers. For considered in this paper example, one MC step with ANN-predicted energy using Model 1 is by a factor of about 5–10 more time-consuming than computing energy by pair potentials. Model 2 is additionally twice as computationally intensive as Model 1, using three times as many structural descriptors. It is likely, however, that the method can be computationally feasible for substantially larger and more complicated systems with multiple CG sites. Furthermore, we note that trained models can be plugged into any molecular dynamics software designed to work with Behler–Parrinello ANN, for example, the N2P2 module of LAMMPS.^67^

Further efforts in finding the optimal neural network hyperparameters and better sets of structural descriptors are necessary to improve both the accuracy and transferability of the neural network potentials obtained with the new approach. Furthermore, the inclusion of higher-order symmetry functions with angular terms may help refine the models for the systems with more complicated interactions and different interaction site types. Investigation along these lines will be a matter of our future work.

## Supporting Information Available

Additional information regarding the pretraining setup; Comparisons of the training performance using different sets of symmetry functions and neural network architectures; Heatmaps of ANN model weights; More comparisons for radial distribution functions and angular distributions obtained with IMC and ANN models (PDF)

## Abbreviations

CG: coarse-grained,
RDF: radial distribution function,
IMC: inverse Monte Carlo,
ANN: artificial neural network,
NN: neural network,
ML: machine learning,
MC: Monte Carlo,
PMF: potential of mean force,
MD: molecular dynamics,
ReLU: rectified linear unit
