# Supplementary material for: Coarse-Grained Modeling Using Neural Networks Trained on Structural Data
Source: J Chem Theory Comput. 2023 Sep 15;19(19):6704–17. doi: 10.1021/acs.jctc.3c00516 (PMC10569054; doi:10.1021/acs.jctc.3c00516)
Supplement: Supplementary file 1 — ct3c00516_si_001.pdf [file ct3c00516_si_001.pdf]

Supporting Information
Coarse-Grained Modeling using Neural Networks Trained on Structural Data

Mikhail Ivanov, Maksim Posysoev, and Alexander P. Lyubartsev*

Department of Materials and Environmental Chemistry, Stockholm University, SE-106 91, Stockholm, Sweden

E-mail: alexander.lyubartsev@mmk.su.se

Phone: +46 8 161193

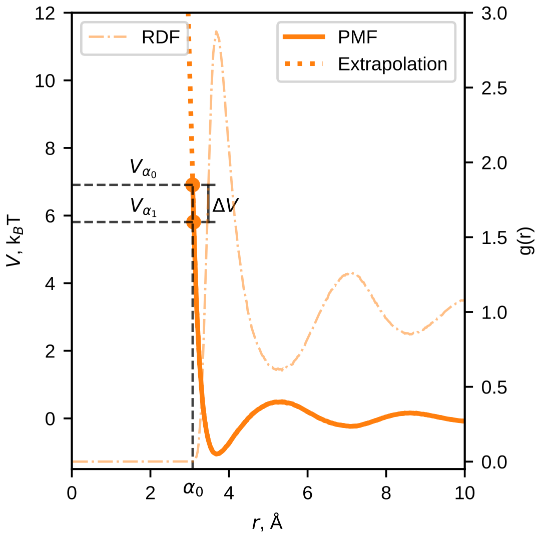

Figure S1: Smooth potential of mean force for liquid argon at 95 K as well as the corresponding RDF.

See G^2 symmetry function set description in Table S1.

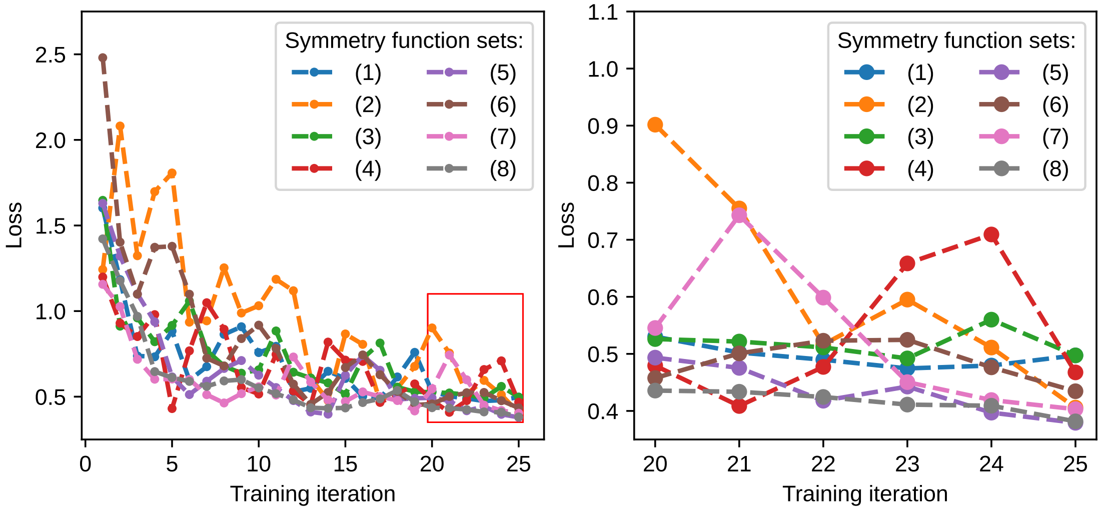

Figure S2: RDF losses of linear models with different sets of G^2 symmetry functions.

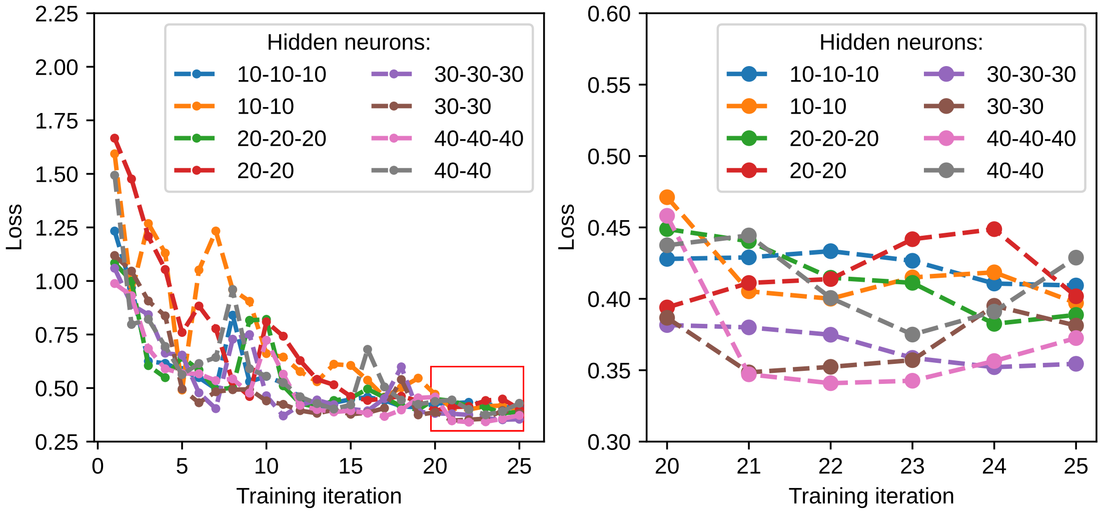

Figure S3: RDF losses for different neural network architectures using the symmetry function set (2).

Table S1: G^2 symmetry function sets.

**Set 1 (Model 1)** $N(G^2) = 8$			**Set 2 (Model 2)** $N(G^2) = 24$			**Set 3** $N(G^2) = 13$			**Set 4** $N(G^2) = 19$		
η, Å^{-2}	r_c, Å	r_s, Å	η, Å^{-2}	r_c, Å	r_s, Å	η, Å^{-2}	r_c, Å	r_s, Å	η, Å^{-2}	r_c, Å	r_s, Å
0.125	10.0	0.000	0.125	10.0	0.000	0.125	10.0	0.000	0.125	10.0	0.000
4.000	10.0	3.000	0.020	10.0	0.000	0.020	10.0	0.000	0.020	10.0	0.000
4.000	10.0	3.500	16.000	10.0	3.000	4.000	10.0	3.000	4.000	10.0	3.000
4.000	10.0	4.000	16.000	10.0	3.250	4.000	10.0	3.500	4.000	10.0	3.500
4.000	10.0	4.500	16.000	10.0	3.500	4.000	10.0	4.000	4.000	10.0	4.000
4.000	10.0	5.000	16.000	10.0	3.750	4.000	10.0	4.500	4.000	10.0	4.500
4.000	10.0	5.500	16.000	10.0	4.000	4.000	10.0	5.000	4.000	10.0	5.000
4.000	10.0	6.000	16.000	10.0	4.250	4.000	10.0	5.500	4.000	10.0	5.500
			16.000	10.0	4.500	4.000	10.0	6.000	4.000	10.0	6.000
			16.000	10.0	4.750	4.000	10.0	6.500	4.000	10.0	6.500
			16.000	10.0	5.000	4.000	10.0	7.000	4.000	10.0	7.000
			4.000	10.0	3.000	4.000	10.0	7.500	4.000	10.0	7.500
			4.000	10.0	3.500	4.000	10.0	8.000	4.000	10.0	8.000
			4.000	10.0	4.000				2.000	10.0	3.000
			4.000	10.0	4.500				2.000	10.0	4.000
			4.000	10.0	5.000				2.000	10.0	5.000
			4.000	10.0	5.500				2.000	10.0	6.000
			4.000	10.0	6.000				2.000	10.0	7.000
			2.000	10.0	3.000				2.000	10.0	8.000
			2.000	10.0	4.000						
			2.000	10.0	5.000						
			2.000	10.0	6.000						
			2.000	10.0	7.000						
			2.000	10.0	8.000						

**Set 5** $N(G^2) = 26$			**Set 6** $N(G^2) = 33$			**Set 7** $N(G^2) = 27$			**Set 8** $N(G^2) = 33$		
η, Å^{-2}	r_c, Å	r_s, Å	η, Å^{-2}	r_c, Å	r_s, Å	η, Å^{-2}	r_c, Å	r_s, Å	η, Å^{-2}	r_c, Å	r_s, Å
0.125	10.0	0.000	0.125	10.0	0.000	0.125	10.0	0.000	0.125	10.0	0.000
0.020	10.0	0.000	0.020	10.0	0.000	0.020	10.0	0.000	0.020	10.0	0.000
16.000	10.0	3.000	16.000	10.0	3.000	16.000	10.0	3.000	16.000	10.0	3.000
16.000	10.0	3.250	16.000	10.0	3.125	16.000	10.0	3.125	16.000	10.0	3.125
16.000	10.0	3.500	16.000	10.0	3.250	16.000	10.0	3.250	16.000	10.0	3.250
16.000	10.0	3.750	16.000	10.0	3.375	16.000	10.0	3.375	16.000	10.0	3.375
16.000	10.0	4.000	16.000	10.0	3.500	16.000	10.0	3.500	16.000	10.0	3.500
16.000	10.0	4.250	16.000	10.0	3.625	16.000	10.0	3.625	16.000	10.0	3.625
16.000	10.0	4.500	16.000	10.0	3.750	16.000	10.0	3.750	16.000	10.0	3.750
16.000	10.0	4.750	16.000	10.0	3.875	16.000	10.0	3.875	16.000	10.0	3.875
16.000	10.0	5.000	16.000	10.0	4.000	16.000	10.0	4.000	16.000	10.0	4.000
16.000	10.0	5.250	8.000	10.0	3.000	8.000	10.0	3.000	8.000	10.0	3.000
16.000	10.0	5.500	8.000	10.0	3.250	8.000	10.0	3.250	8.000	10.0	3.250
16.000	10.0	5.750	8.000	10.0	3.500	8.000	10.0	3.500	8.000	10.0	3.500
16.000	10.0	6.000	8.000	10.0	3.750	8.000	10.0	3.750	8.000	10.0	3.750
4.000	10.0	3.000	8.000	10.0	4.000	8.000	10.0	4.000	8.000	10.0	4.000
4.000	10.0	3.500	8.000	10.0	4.250	8.000	10.0	4.250	8.000	10.0	4.250
4.000	10.0	4.000	8.000	10.0	4.500	8.000	10.0	4.500	8.000	10.0	4.500
4.000	10.0	4.500	8.000	10.0	4.750	8.000	10.0	4.750	8.000	10.0	4.750
4.000	10.0	5.000	8.000	10.0	5.000	8.000	10.0	5.000	8.000	10.0	5.000
4.000	10.0	5.500	4.000	10.0	3.000	4.000	10.0	3.000	8.000	10.0	3.250
4.000	10.0	6.000	4.000	10.0	3.500	4.000	10.0	3.500	8.000	10.0	3.500
4.000	10.0	6.500	4.000	10.0	4.000	4.000	10.0	4.000	8.000	10.0	3.750
4.000	10.0	7.000	4.000	10.0	4.500	4.000	10.0	4.500	8.000	10.0	4.000
4.000	10.0	7.500	4.000	10.0	5.000	4.000	10.0	5.000	4.000	10.0	3.000
4.000	10.0	8.000	4.000	10.0	5.500	4.000	10.0	5.500	4.000	10.0	3.500
			4.000	10.0	6.000	4.000	10.0	6.000	4.000	10.0	4.000
			2.000	10.0	3.000				4.000	10.0	4.500
			2.000	10.0	4.000				4.000	10.0	5.000
			2.000	10.0	5.000				4.000	10.0	5.500
			2.000	10.0	6.000				4.000	10.0	6.000
			2.000	10.0	7.000				2.000	10.0	7.000
			2.000	10.0	8.000				2.000	10.0	8.000

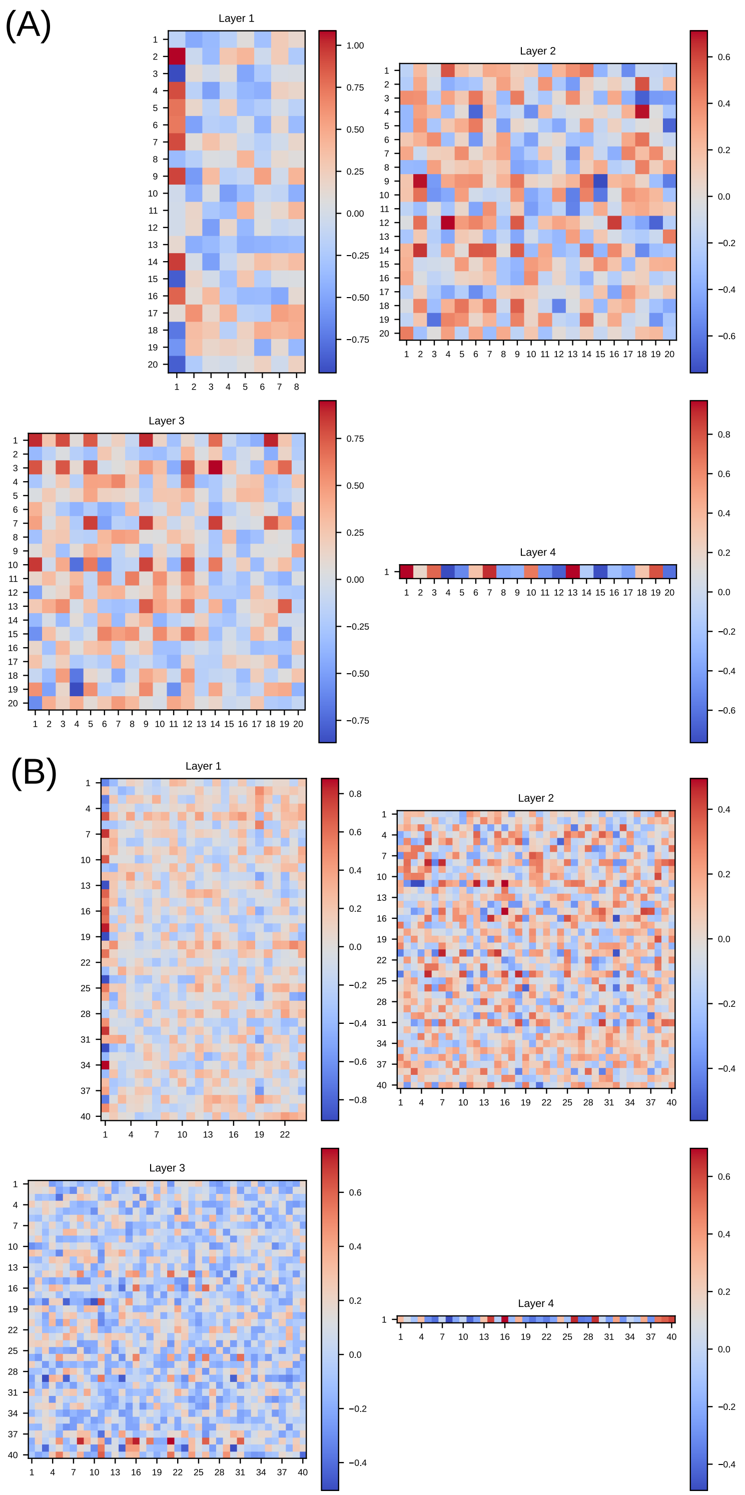

Figure S4: Weight parameters of the neural network potentials for CG methanol Model 1 (A) and Model 2 (B).

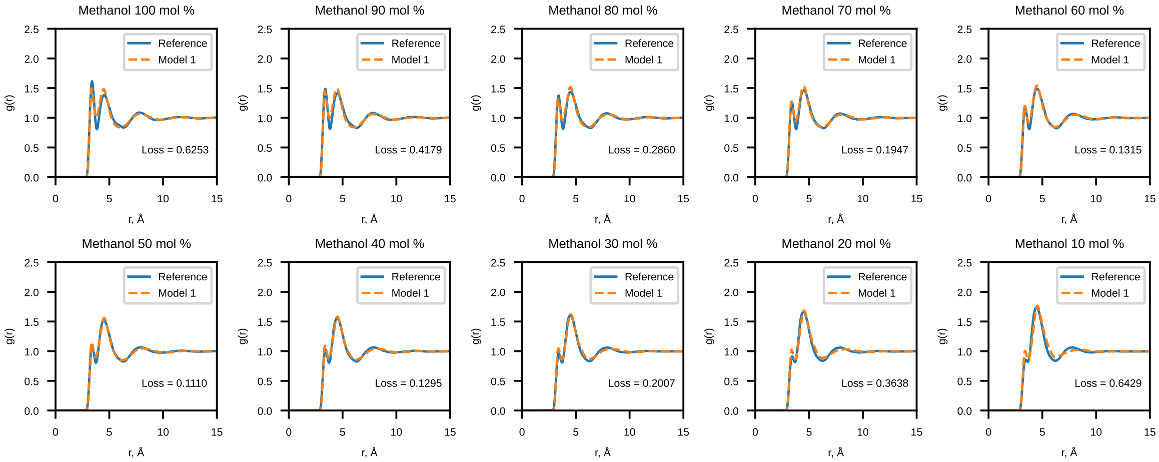

Figure S5: Comparison of the reference RDF and the RDF sampled with Model 1 for CG water-methanol mixtures.

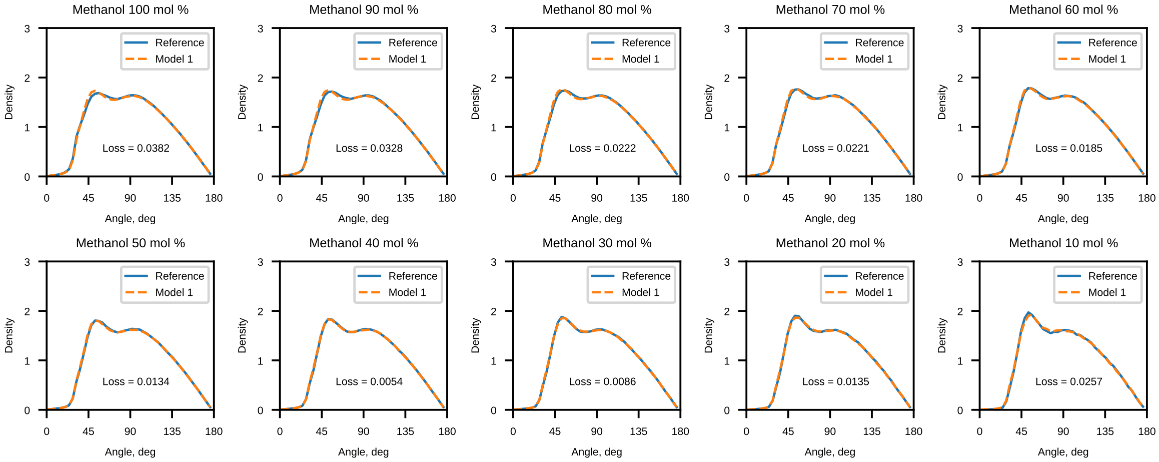

Figure S6: Comparison of the reference angular distribution and the distribution sampled with Model 1 for CG water-methanol mixtures.

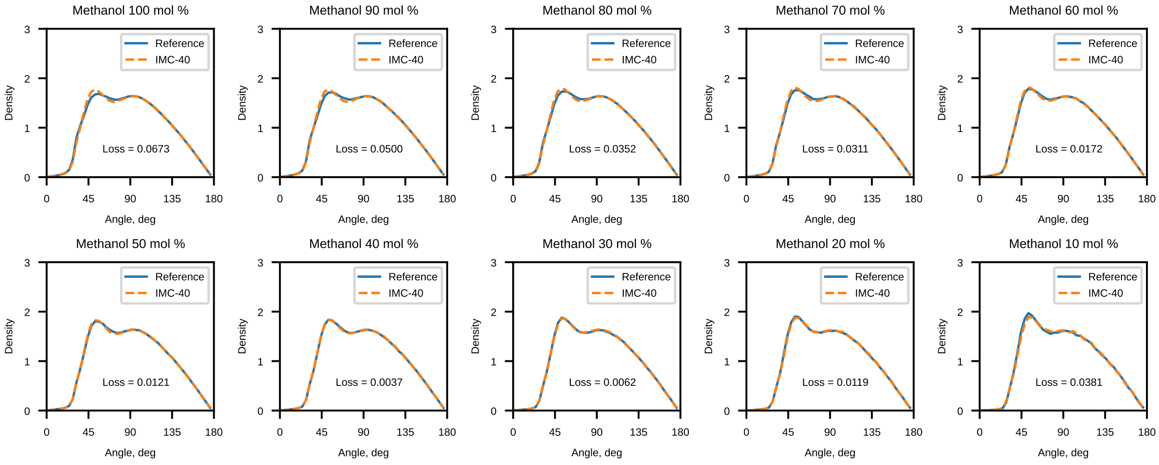

Figure S7: Comparison of the reference angular distribution and the distribution sampled with the IMC effective potential trained on 40 mol% methanol.

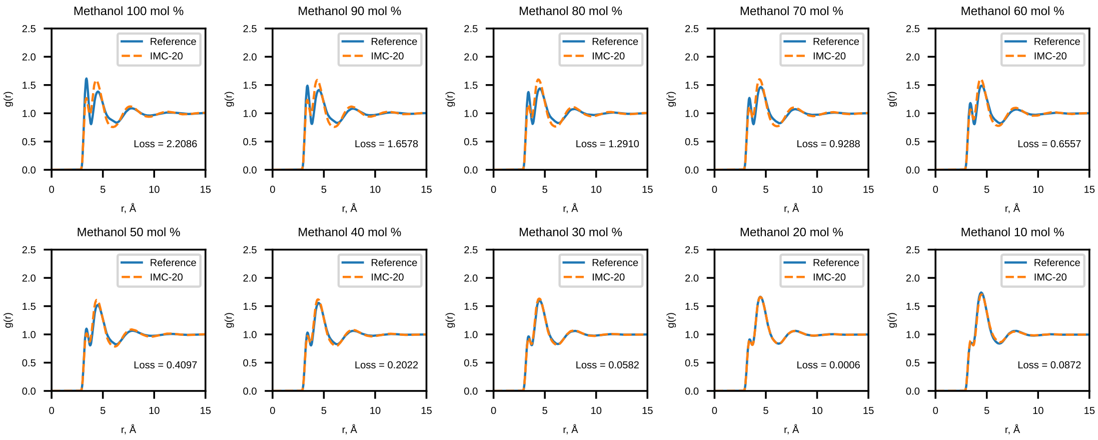

Figure S8: Comparison of the reference RDF and the RDF sampled with the IMC effective potential trained on 20 mol% methanol.

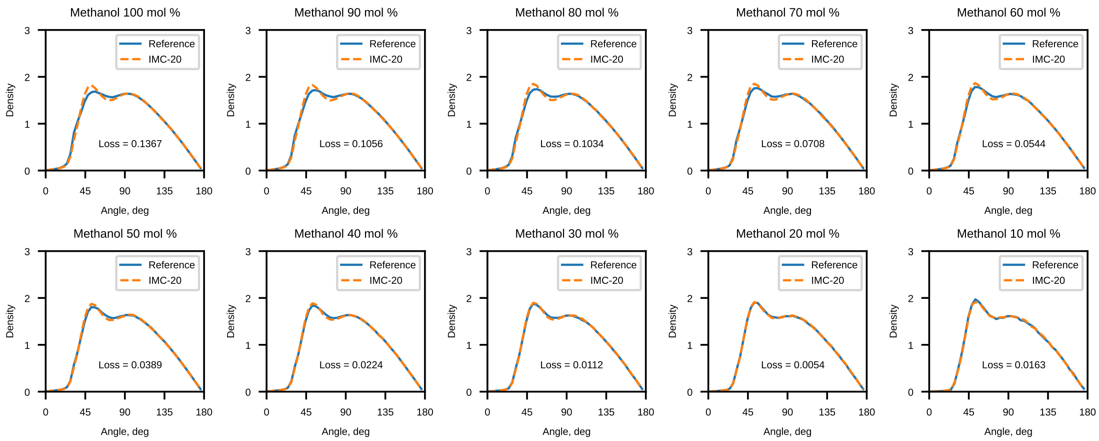

Figure S9: Comparison of the reference angular distribution and the distribution sampled with the IMC effective potential trained on 20 mol% methanol.

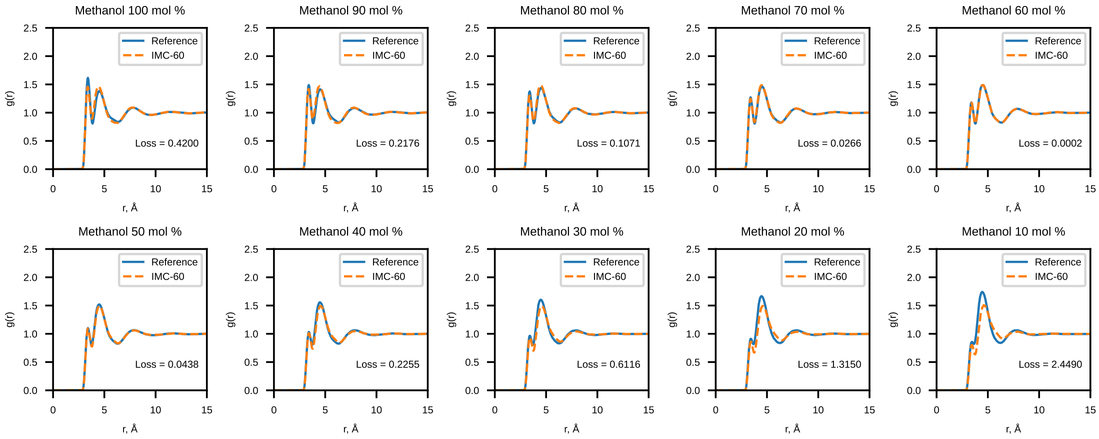

Figure S10: Comparison of the reference RDF and the RDF sampled with the IMC effective potential trained on 60 mol% methanol.

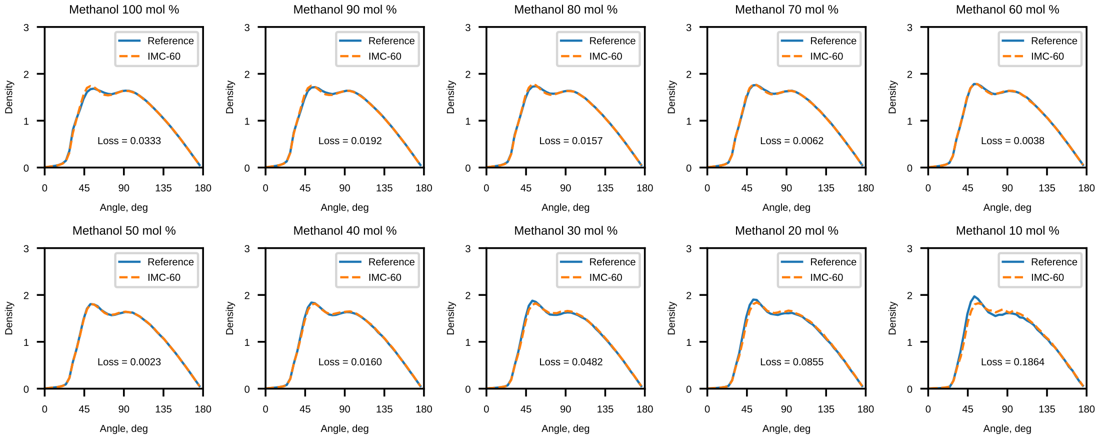

Figure S11: Comparison of the reference angular distribution and the distribution sampled with the IMC effective potential trained on 60 mol% methanol.

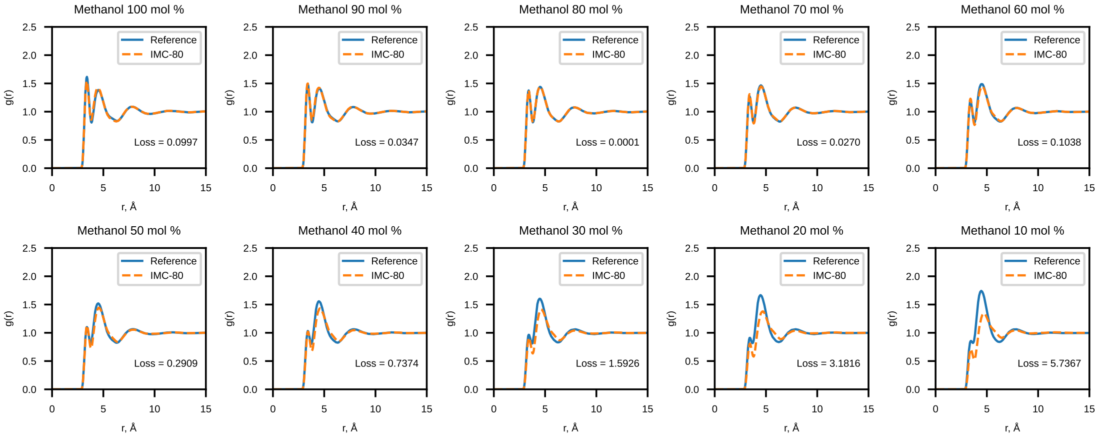

Figure S12: Comparison of the reference RDF and the RDF sampled with the IMC effective potential trained on 80 mol% methanol.

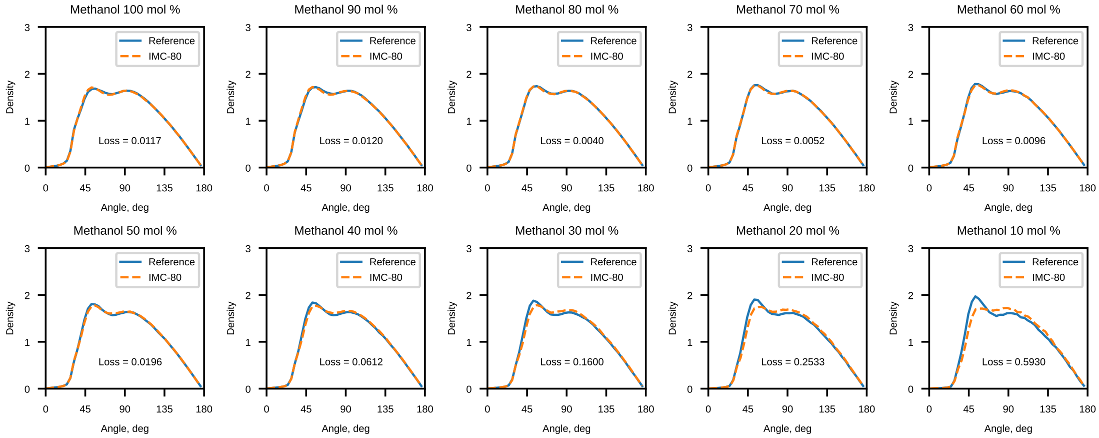

Figure S13: Comparison of the reference angular distribution and the distribution sampled with the IMC effective potential trained on 80 mol% methanol.

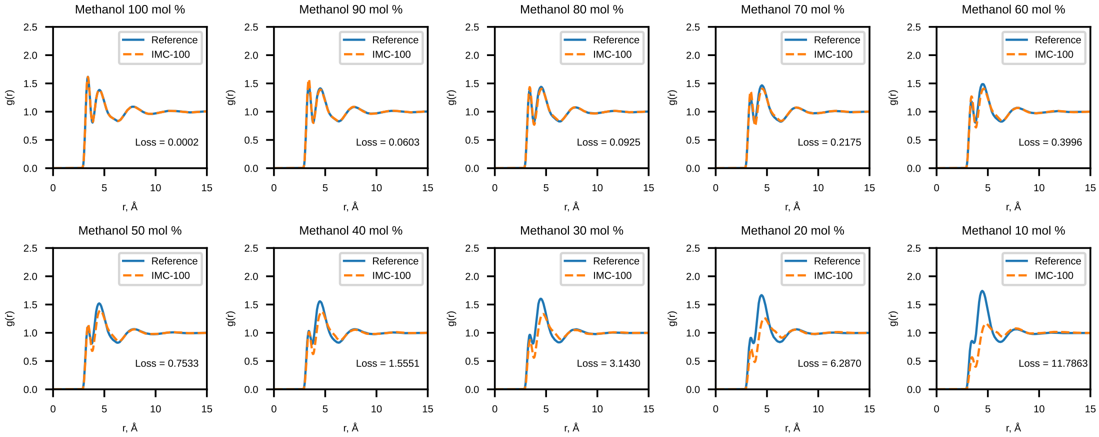

Figure S14: Comparison of the reference RDF and the RDF sampled with the IMC effective potential trained on 100 mol% methanol.

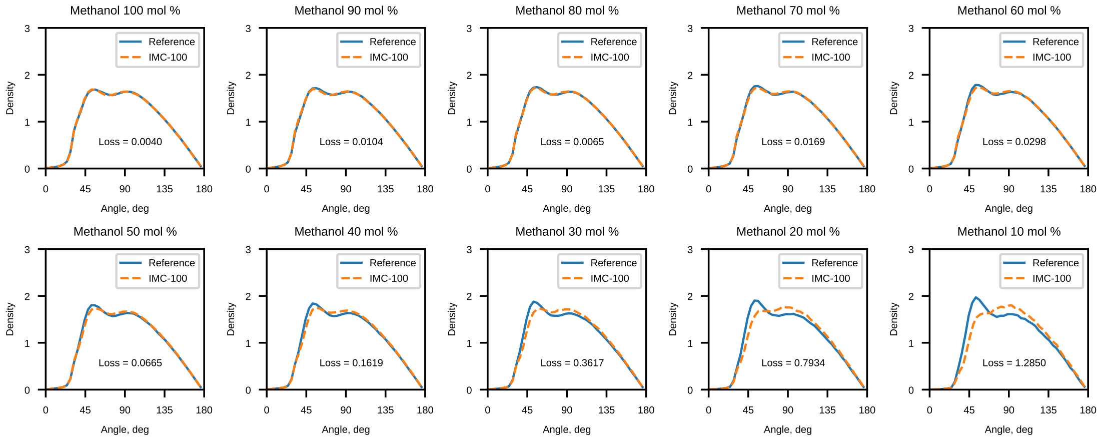

Figure S15: Comparison of the reference angular distribution and the distribution sampled with the IMC effective potential trained on 100 mol% methanol.

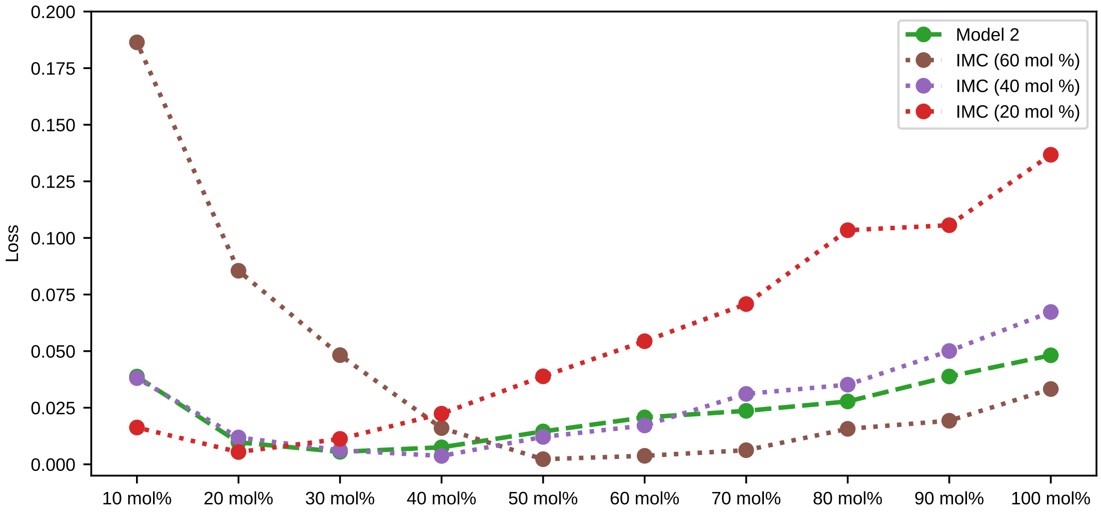

Figure S16: Comparison of the angular distribution loss values obtained for Model 2 and three of the best IMC models.
